# Current trends and challenges in cancer management and therapy using designer nanomaterials

**DOI:** 10.1186/s40580-019-0193-2

**Published:** 2019-07-15

**Authors:** P. N. Navya, Anubhav Kaphle, S. P. Srinivas, Suresh Kumar Bhargava, Vincent M. Rotello, Hemant Kumar Daima

**Affiliations:** 1 Nano-Bio Interfacial Research Laboratory (NBIRL), Department of Biotechnology, Siddaganga Institute of Technology, Tumkur, Karnataka 572103 India; 2 Melbourne Integrative Genomics, School of BioSciences/School of Mathematics and Statistics, The University of Melbourne, Melbourne, VIC 3010 Australia; 3 School of Optometry, Indiana University, Bloomington, Indiana 47405 USA; 4 Centre for Advanced Materials and Industrial Chemistry, School of Science, RMIT University, Melbourne, VIC 3001 Australia; 5 Department of Chemistry, University of Massachusetts (UMass) Amherst, 710 North Pleasant Street, Amherst, MA 01003 USA; 6 Amity Institute of Biotechnology, Amity University Rajasthan, Kant Kalwar, NH-11C, Jaipur-Delhi Highway, Jaipur, Rajasthan 303002 India; 7 Department of Biotechnology, Bannari Amman Institute of Technology, Sathyamangalam, Erode, Tamil Nadu 638401 India

**Keywords:** Drug delivery, Cancer therapy, Engineered nanomaterials, Next-generation, Nanotoxicity

## Abstract

Nanotechnology has the potential to circumvent several drawbacks of conventional therapeutic formulations. In fact, significant strides have been made towards the application of engineered nanomaterials for the treatment of cancer with high specificity, sensitivity and efficacy. Tailor-made nanomaterials functionalized with specific ligands can target cancer cells in a predictable manner and deliver encapsulated payloads effectively. Moreover, nanomaterials can also be designed for increased drug loading, improved half-life in the body, controlled release, and selective distribution by modifying their composition, size, morphology, and surface chemistry. To date, polymeric nanomaterials, metallic nanoparticles, carbon-based materials, liposomes, and dendrimers have been developed as smart drug delivery systems for cancer treatment, demonstrating enhanced pharmacokinetic and pharmacodynamic profiles over conventional formulations due to their nanoscale size and unique physicochemical characteristics. The data present in the literature suggest that nanotechnology will provide next-generation platforms for cancer management and anticancer therapy. Therefore, in this critical review, we summarize a range of nanomaterials which are currently being employed for anticancer therapies and discuss the fundamental role of their physicochemical properties in cancer management. We further elaborate on the topical progress made to date toward nanomaterial engineering for cancer therapy, including current strategies for drug targeting and release for efficient cancer administration. We also discuss issues of nanotoxicity, which is an often-neglected feature of nanotechnology. Finally, we attempt to summarize the current challenges in nanotherapeutics and provide an outlook on the future of this important field.

## Introduction

Cancer is one of the foremost causes of death globally. Despite efforts to mitigate risk factors in recent decades, the prevalence of cancer is continuing to increase [1]. Current standards of care combine precise staging of cancer with chemotherapy, radiotherapy, and/or surgical resection. Radiotherapy and chemotherapy are known for significant adverse effects [2], with most methods targeting non-specifically any rapidly dividing cells irrespective of whether they are tumorous or not. Furthermore, poor pharmacokinetic characteristics of anticancer drugs arising from poor solubility, stability, and metabolism pose different challenges of toxicity, inefficacy and limited bio-distribution. Thus, it is imperative to develop effective formulations that can address the above cited challenges and provide selective targeting of tumor sites without significant damage to the viability of healthy tissues [3–9].

**Fig. 1:**
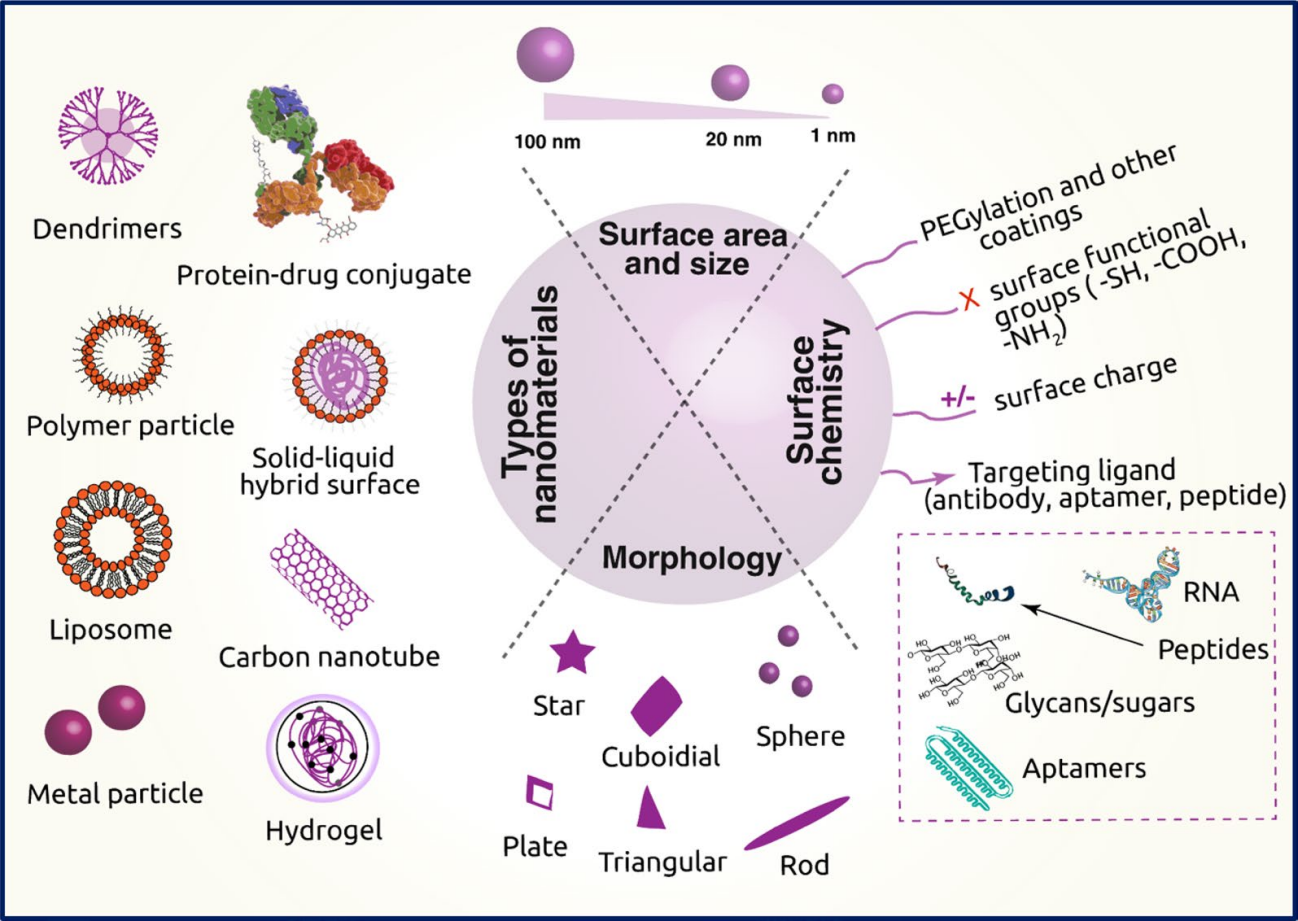
Schematic representation of different types of nanomaterials employed in cancer therapy, their important physical properties and surface chemistry required to carry drugs

In the paradigm of ‘nanomedicine’, nanotechnology is being embraced to obtain effective drug delivery, establish novel in vitro diagnostics, and develop nano-based implants [7, 10, 11]. There is an exponential growth in the field of nano-based sensing and drug delivery [12–20]. Nano-based modalities provide enhanced transport across biological barriers, enable selective targeting of malignant tissues/cells, and offer strategies for sustained release of a drug [21, 22]. As illustrated in Fig. 1, a wide range of nanomaterials have been fabricated using organic, inorganic, lipid and protein compounds typically in the range of 1–100 nm and deliver various antitumor drugs by fine-tuning the chemical composition, size, and shape (morphology) that can control the functionality of the nanomaterials. Specifically, the use of nanocarriers for drug delivery offers many advantages; (i) circumvent the problems of solubility and stability of anticancer drugs; (ii) prevents the drug from degradation from proteases and other enzymes and increase the half-life of the drug in the systemic circulation; (iii) improves drug distribution and targeting; (iv) helps in the sustained release of drug by targeting the cancer sites and (v) helps in delivery of multiple drugs and, therefore helps in reducing drug resistance [23]. Thus, nanotechnology is creating new opportunities for designing materials that can revolutionize the approaches to drug delivery and transform the landscape of the pharmacological treatment of cancer [7, 24–26].

In this review, we discuss the development of ‘smart’ nanomaterials for treating cancer, with emphasis on the strategies of drug targeting and triggering sustained release of drug from the nanocarriers. Later, we elaborate upon the design and fabrication of nanomaterials, along with different types of nanomaterials used in cancer therapeutics including liposomes, dendrimers, inorganic nanomaterials and polymeric nanomaterials. We also discuss the current challenges and perspectives of nanomaterials in effective cancer management.

## Approaches for drug vehicles, targeting, and release

It is well-known that the activity of the anticancer drugs is greatly attenuated by the time drug reaches the target, which can render the treatment to be ineffective and increase off-target effects. The effectiveness of anticancer drug treatment can be achieved only when the administered drug is of proper dosage and display maximal activity in the cancer cells. Thus, the nanomaterials used for targeting tumor cells should have the capability of increasing local concentration of the drugs in and around tumor cells, thereby reducing the potential toxicity toward healthy cells [27]. The efficient delivery of nanomaterials to the target tissues can be classified as passive and active targeting, as discussed below.

### Passive targeting

**Fig. 2:**
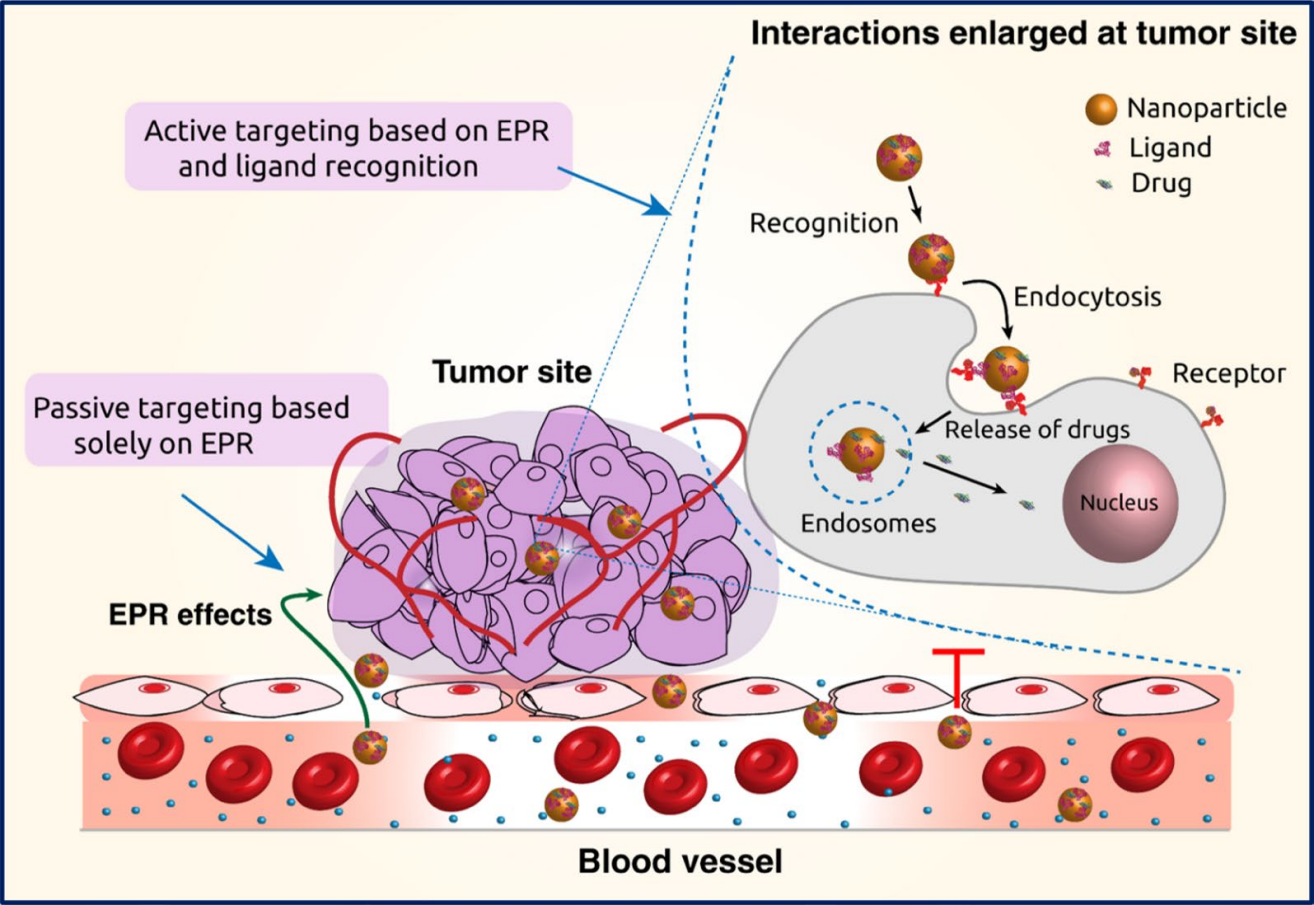
Graphical illustration of passive and active drug targeting strategies. In passive targeting, the nanocarriers pass through the leaky walls and accumulate at the tumor site by the enhanced permeability and retention (EPR) effect. Active targeting can be achieved using specific ligands that bind to the receptors on the tumor cells

The most common route of administration of nanomaterial-based anticancer drugs is intravenous injection. This approach bypasses the absorption step across the intestinal epithelium required after oral administration [28]. At tumor sites, the vascular barrier is disrupted, and this enables nanocarriers to accumulate in the tumor tissue as depicted in Fig. 2 [29]. The gaps between the endothelial cells in the tumor vasculature can range from 200 to 2000 nm depending on the tumor type, localization, and environment. Moreover, due to the poor lymphatic function, the nanoparticles are not rapidly cleared and accumulate in the tumor interstitium [30]. This is known as enhanced permeability and retention (EPR) effect, which is the basis of passive targeting [31]. This accumulation of the drug at the tumor sites is a passive process, and it requires prolonged circulation of the drug for appropriate drug delivery. The accumulation of the nanocarriers is essentially depends on physicochemical properties such as size, shape (morphology), surface charge and surface chemistry [32]. The extent and kinetics of nanomaterial accumulation at the tumor site are influenced by their size. The nanocarriers need to be smaller than the cut-off of the proportions in the neovasculature, with the extravasation to the tumor acutely affected by the size of the vehicle. Further, the biodistribution of the nanomaterial–drug formulation is influenced by blood perfusion, passive interactions with biomolecules along the route, and immunological clearance processes such as phagocytosis or renal clearance [33].

There are several studies reporting on successful applications of passive targeting of tumor cells and a successful translation into clinical therapeutics. The first FDA (the Food and Drug Administration, national agency of the United States Department of Health and Human Services) approved nano-drug is one consisting of PEGylated liposome entrapped doxorubicin (DOX) targeted against HIV-related Kaposi sarcoma tumor, and ovarian cancer. Entrapping doxorubicin inside the lipid material resulted in substantial reduction in the cellular and systemic toxicity of the drug, and resulted in improved pharmacokinetics for the drug, controlled biodistribution, and release [34]. A recent FDA-approved nano-formulation comprising of liposomal entrapped cytarabine–daunorubicin combination (CPX-351 Vyxeos™) has shown 9.6 months of overall survival compared with 6.0 months of survival for the free form of the drug in patients with newly diagnosed high-risk acute myeloid leukemia [35]. Many such formulations have been approved [34], opening new avenues toward cancer therapeutics.

An additional layer of targeting functionalities can be applied to these nano-formulations to improve their biodistribution and minimize immune clearance. The concept has been discussed in the active targeting section of this review. Therapeutic efficacy of passive targeted approaches is limited by the heterogeneity of the EPR effects seen within and between different tumors. Due to variable endothelial gaps resulting from vigorous tumorous cell growth, it can result in non-uniform extravasation of nanoparticles into the target area [36]. Additionally, while it is evident that nanoparticles permeability should normally be at higher rates in hypoxic core of tumor area rather than the periphery, few studies contrast this observation [37]. This heterogeneity adds another layer of complexity to passive targeting. Similarly, extravasation has been shown to not only depend on permeability, but also on the blood flow rate around the tumor site. This vascularization displays spatial and temporal heterogeneity within and between tumor cells adding another level of challenges to passive targeting [38]. Moreover, for nanomaterials that do extravasate by crossing the vasculature, deeper penetration to tumor site is impeded by the interstitial tumor matrix. Here, the size and size-dependent properties of the material will be the key to improving penetration into the matrix. Wong et al. have proposed a multi-factorial nanosystem that changes size upon reaching different locations of the tumor sites. They have developed gelatin particles, 100 nm in diameter, which upon extravasation into tumor tissue reduce in size to 10 nm, through degradation by tumor-associated matrix metalloproteinases [39].

Unfortunately, the understanding of EPR effects is limited by the unavailability of accurately recapitulated solid tumor models in humans. In fact, most of our current knowledge is based on a few subcutaneous tumor xenograft models that divide vigorously resulting in very high EPR effects. Therefore, the knowledge from experimentation using these models could provide a false impression about the efficacy of passive targeted nanomaterials [40]. Moreover, it is imperative to state that there is also a lack of patient-based experimental data on the EPR phenomenon. Therefore, further advances in understanding tumor biology, understanding EPR effects in varieties of the tumor is essential. Such thoughtful knowledge will be useful in the rational tailoring of nanomaterials, which can be used for personalized tumor medicine for even higher therapeutic benefits.

### Active targeting

Active targeting, also known as the ligand-mediated targeted approach, involves affinity based recognition, retention and facilitated uptake by the targeted cells (Fig. 2) [32]. Chemical affinity for active targeting is based on different specific molecular interactions such as receptor–ligand-based interactions, charge-based interactions and facilitated motif-based interactions with substrate molecules [41, 42]. Diverse biomolecules can constitute a ligand, including antibodies, proteins, nucleic acids, peptides, carbohydrates and small organic molecules such as vitamins [43–45]. Target substrates can be surface molecules expressed in diseased cells, proteins, sugars or lipids present in the organs, molecules present (or secreted by tumor cells) in the microenvironment of the diseased cells or even the physicochemical environment in the vicinity [46]. Nanomaterial-based smart, targeted systems exploit the multivalent nature of interactions of ligands with the target antigens. When multiple ligand molecules are accumulated onto the nanosystems, there is an overall increase in the avidity of the nanoparticles for its cognate target [45]. Also, binding of one ligand molecule generally facilitates binding of consequent molecules through cooperativity effects, collectively enhancing the binding efficiency and subsequent actions.

**Fig. 3:**
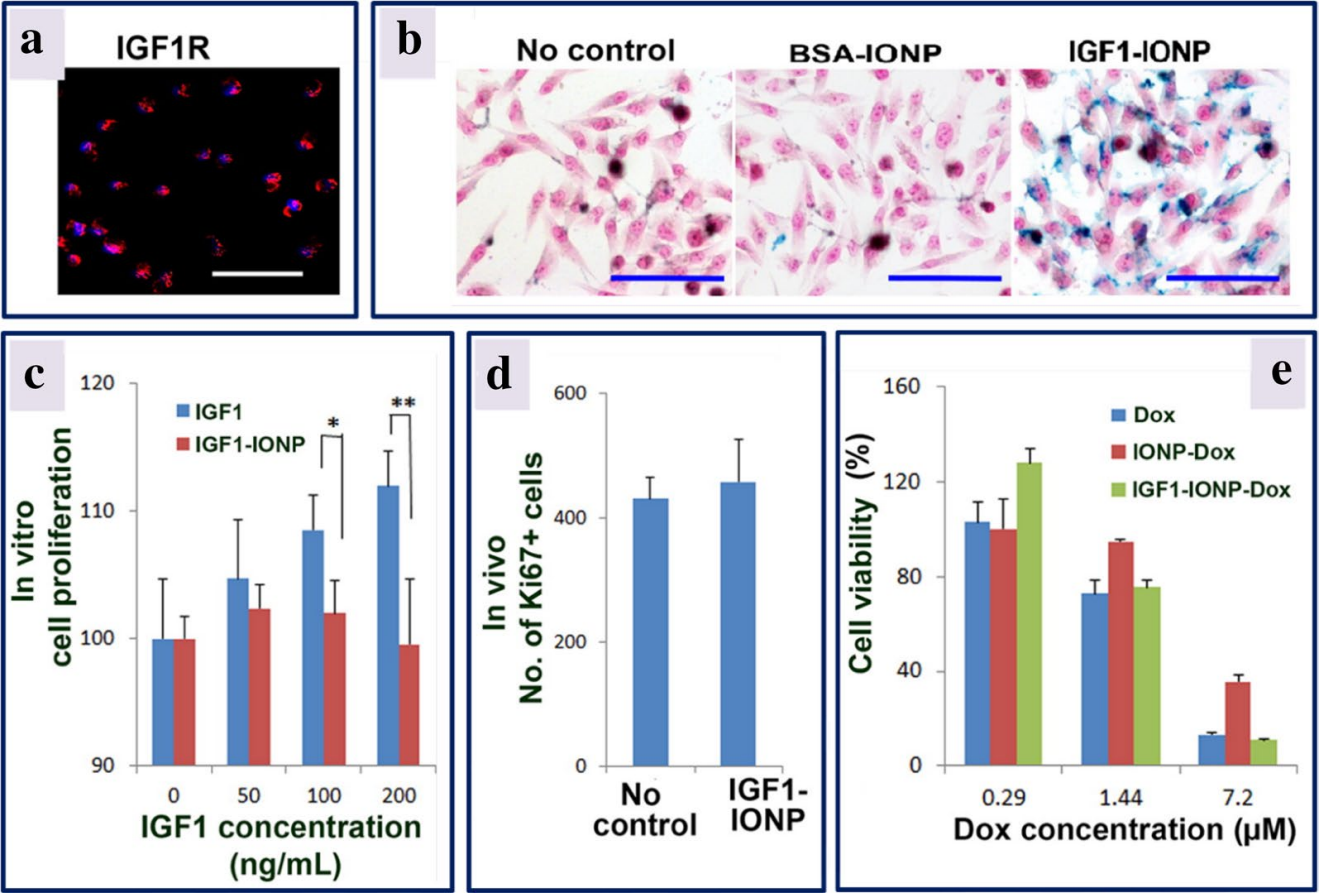
In vitro and in vivo effects of IGF1-IONPs (insulin-like growth factor 1-iron oxide nanoparticles) and IGF1-IONPs-doxorubicin on cell proliferation and viability. **a** The effect of IGF1R in MIAPaCa-2 cells was assessed by immunofluorescence labeling employing an anti-IGF1R antibody (shown in red color). **b** Prussian blue staining of cells incubated for 4 h with different treatments at 20 μg/mL of iron equivalent dose. The cells are also counterstained with nuclear fast red. **c** The in vitro influence of IGF1 and IGF1-IONPs on cell proliferation. The % of viable cells after 96 h incubation with IGF1 or IGF1-IONPs, and for 4 h at equivalent IGF1 concentrations was estimated by cell proliferation assay, wherein *P < 0.05; **P < 0.001. **d** The in vivo effect on tumor cell proliferation of IGF1-IONPs in human pancreatic PDX-tumor xenografts. By using immunofluorescence labeling of an anti-Ki67 antibody, the Ki67-positive cells in tumor sections after two tail vein injections of 20 mg/kg iron dose of IGF1-IONPs are measured. **e** In vitro cytotoxicity of unconjugated and conjugated doxorubicin in MIA PaCa-2 cells. The scale bars are 100 μm (adapted with permission from [48])

Active targeting approach has been exploited to increase internalization of nanoparticles by the target cells and improve the drug delivery efficacy. In one study, anti-HER2 targeting ligand moieties functionalized on the surface of liposome increased the cellular uptake of the nanoparticles in HER2-expressing cancer cells. In contrast, non-HER2 targeting moieties or non-targeted liposome nanoparticles resulted in the accumulation of particles in the perivascular and stromal space of the tumor site in higher proportion. These accumulated nanoparticles were captured and quickly cleared by macrophages resulting in suboptimal tumor cell internalization [47]. In another study, Zhou et al. [48] synthesized insulin-like growth factor 1 (IGF1) and conjugated it to magnetic iron oxide nanoparticles (IONPs) having the anthracycline doxorubicin as therapeutic payload. Upon intravenous administration of nanoparticles into patient-derived xenograft (PDX) prototype of pancreatic cancer, exceptional tumor targeting and penetration was obtained. Targeted therapy using theranostic IGF1-iron oxide nanoparticles-doxorubicin significantly inhibited the growth of pancreatic PDX tumors showing potential for improved therapeutic outcomes as shown in Fig. 3. Further, they measured the localization and internalization of these nanoparticles using magnetic resonance imaging (MRI) exploiting IONPs properties as contrast agents. These targeted magnetic nanosystems could also be used in photothermal therapy, wherein, their specific localization in tumor sites can be used to induce a local thermal ablation of the tumor sites upon passing alternating magnetic field (AMF). The heat generated due to Neel and Brownian relaxation as well as hysteresis loss can be used to kill tumor cells in the vicinity of IONPs [49].

Usually, targeting based approaches exploit the subtle differences in the expression of substrate molecules between cancer and normal cells. For example, epidermal growth factor receptor (EGFR, responsible for epithelial tissue development and homeostasis) is overexpressed in cancerous cells relative to normal cells, as cancer cells grow and divide vigorously [50]. This concentration difference on the cell surface is the basis for studies targeting cancer cells overexpressing EGFR [51, 52]. Normally, given the complexity of nanoparticles administration routes and undesirable interactions with non-specific molecules within the organisms, the difference in the nanoparticle’s affinity towards cancerous and normal cells would not be sufficient for high specificity and efficient delivery to the target site required for wide utility for biomedical applications. Recent approaches have explored concomitantly targeting multiple surface receptors with single nanoparticle systems conjugated with multiple ligands [53]. Bhattacharyya et al. have fabricated and characterized such dual ligand–receptor nanosystems using gold (Au) nanoparticles. They employed EGFR and folate receptor (FR) overexpressed in ovarian cancer as target surface molecules, and used monoclonal antibodies against these receptors as dual ligands for Au nanoparticle targeting. They observed that this dual targeting system is more efficient in delivering Au nanoparticles to cancer cells than their corresponding single ligand system [54].

However, there are multiple factors that need to be optimized for effective use of active-targeted cancer therapeutics. Ligand density on the nanoparticles dictates the strength of avidity towards the substrate, so approaches used to conjugate ligands on the surface of nanoparticles are critical aspects of the targeted systems. Generally, covalent conjugation methods have been utilised, but systems with physical absorption using affinity complexes can also be used effectively [55]. The critical aspect to this conjugation is to maintain the stability of the conjugated ligands during the adverse environment presented by the physiological environment, and various approaches have been undertaken to achieve it [32]. Interestingly, in contrast to the more-ligand-more-targeting notion, there have been a few observations wherein increasing ligand density to increase total affinity did not always have a linear relationship with ligand density. This phenomenon has been explained based on molecular saturation, improper orientation of ligands, bond constraints, and steric constraints from neighboring molecules on the nanoparticles [56]. Similarly accumulating a high degree of hydrophobicity on the nanoparticles led to increased susceptibility towards macrophage uptake, without offering a significant advantage for rapid target cell internalization [57]. These studies do raise concerns about how an appropriate optimization of targeting moieties, conjugation approaches and densities play an essential role in the desired outcomes of the therapeutic nanosystems.

Targeting specificity and payload delivery capacity are two critical parameters required to optimize the efficiency and viability of a nanoparticle-based active targeted systems in in vivo settings. Specificity is defined as how effective the interaction is between the ligand-conjugated nanoparticles with their target molecules weighted against off-target effects incurred before reaching the target molecules. This specificity is dictated mostly by the interactions presented during the biodistribution process. Since there are a multitude of smaller interactions presented by diverse complex biomolecules based on simple van der Waals interactions, the cumulative effects of these smaller interactions can hinder nanoparticles approach to their target sites. Similarly, in an in vivo environment, many smaller proteins and intrinsic biomolecules bind non-specifically on the surface of nanoparticles, commonly known as Vroman’s effect [58], leading to changed ‘identity’ of the whole nanosystem. This alteration could cause nanoparticles to lose their specificity leading to sub-optimal localization in desired sites or at cellular targets. Since actively-targeted nanosystem rely on being in the vicinity of the target sites to home in and execute their functions, biodistribution profile is very critical to its proper functioning. The biodistribution profile is also strongly influenced by active clearance processes posed by various immune cells, and blood flow/renal filtration rate. Since tumor blood flow is low compared to observed in other organs and bodily tissues, the increased affinity based on the ligands cannot compensate for the clearance processes [32]. Therefore, actively-targeted nanosystems need to be developed with extended blood circulation times and biocompatible profiles, along with neutral coating to prevent extensive non-specific binding of blood molecules.

Most cognate substrates for nanoparticles bound ligands are present in the extravascular space of tumor outside of the blood vessels epithelial lining. Active targeting, therefore, relies extensively on endoplasmic retention effects to reach the targets. Therefore, it is essential to consider how we can exploit the endoplasmic retention effects to achieve active targeting. Endoplasmic retention effects vary with tumor types such that some cancers have wide epithelial fenestrations so that nanoparticles with broader size range can be effectively used. However, in some tumor cases the size of nanoparticles should be tuned according to the vasculature lining gap size [59]. Endoplasmic retention is only one of the mechanisms describing tumor biology. There is a multitude of other factors that can present potential challenges for nanotherapeutics such as low blood circulation rate in tumor vessels, tumor site macrophages, and extracellular matrices environment around tumor cells. These factors play significant roles in how targeted nanoparticles find their substrate and effectively deliver drugs payload. A clear understanding of these factors will provide important synthesis strategies for targeted nanoparticles therapy—active or passive targeting alike.

### Drug release strategy

**Fig. 4:**
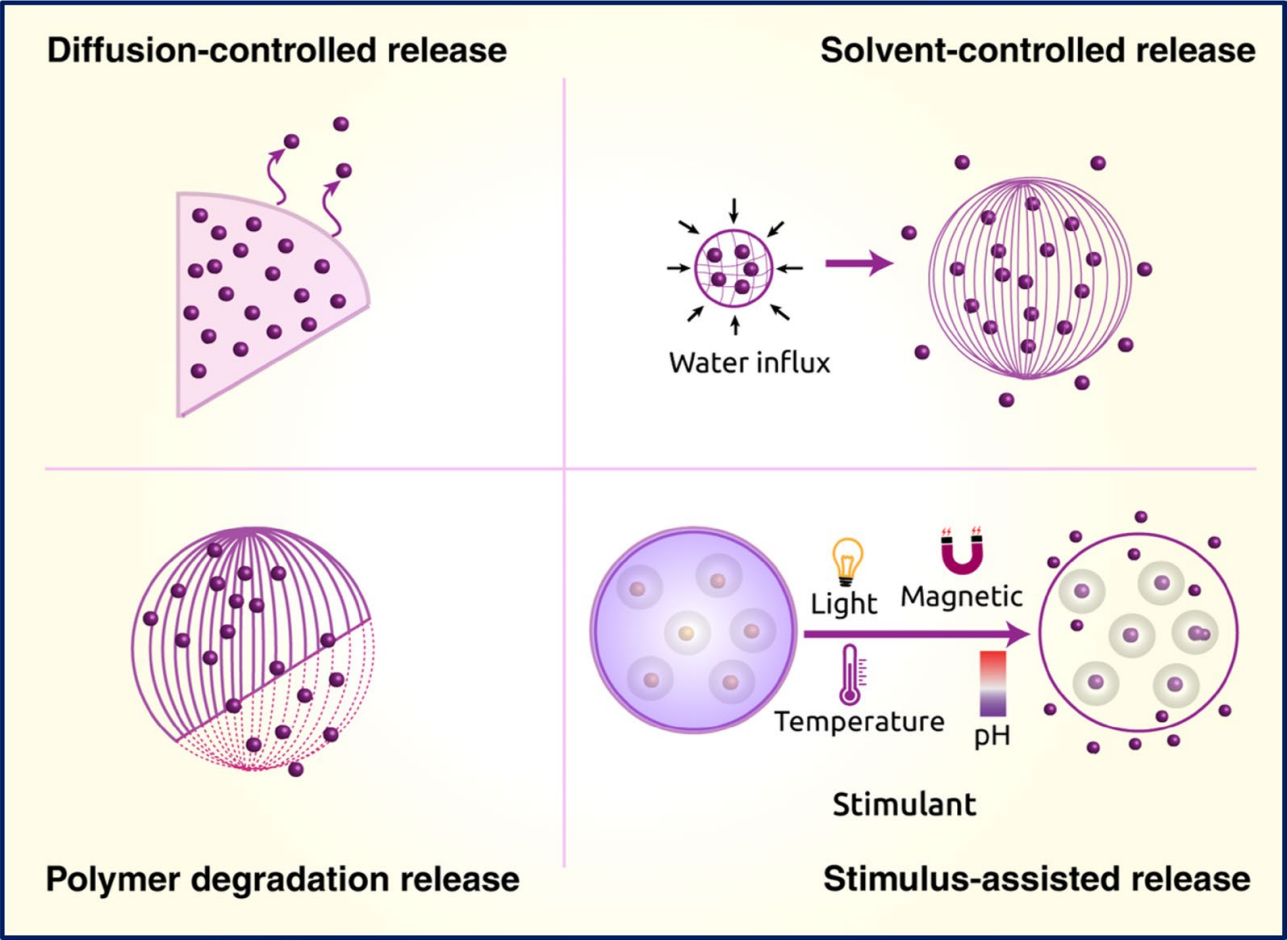
Schematic depiction of diffusion-, solvent-controlled, polymer degradation, and other stimuli reliant drug release

Payload delivery capacity depends on how effectively drugs have been packaged, and how drug release mechanisms are programmed into the nanosystems. Drug ‘packaging’ efficacy depends on encapsulation or drug conjugation efficiency. Different nanoparticles provide different means of entrapping drug molecules, as described later in the section. Modulating rate of drug release in response to an activation signal constitutes an essential strategy to achieve controlled release purposes as well as maintaining effective therapeutic dosage over a stretch of time. There are two categories of nanosystems, open-loop control systems and closed-loop control systems, grouped according to what activation factors stimulate drug release as schematically shown in Fig. 4. In open-loop control systems, external factors such as magnetic pulses, thermal, acoustic pulses or electric fields control drug release. In contrast, in closed-loop systems the drug release rate is controlled by the presence and intensity of internal stimuli in the vicinity of the target sites [60, 61]. A few current strategies are based on the ‘chemistry’ programmed into the nanosystems that are responsive towards pH or temperature, erosion due to the local chemical environment, redox reaction-based release, and enzyme-mediated release as discussed below [62].

#### Redox-activated drug release

In redox-activated drug release mechanism, a redox-responsive nanocarrier containing functional groups that reacts upon contact with oxidizing and/or reducing environment in and around cancer cells (peroxides, GSH, and free radicals), undergoing to chemical bond cleavage [63]. The chemical changes can also introduce changes in the hydrophobicity of the polymer, changing the integrity of nanoparticles and thereby leading to release of drug cargo. For example, in poly(propylene sulfide) polymer nanoparticles, disulfide bonds act as a redox-responsive motif, and upon reacting with H_2_O_2_ leads to a change of hydrophobicity of the polymers causing a collapse of nanoparticles and thus drug release [64]. Redox-response moieties can also respond to the stimuli in a non-linear fashion. This complexity allows a prompt reaction to the high concentration of stimuli, but not to low concentrations, achieving controlled specificity [65].

#### pH-mediated drug release

The extracellular microenvironment of tumor tissues is acidic, due to secreted lactic acid caused by glycolysis in anorexia. Studies show that the pH value drops to around 6.5 from physiological pH of 7.4 during the tumoral metastasis or development [66]. This gradient in the pH profile between pathological cells and normal cells can be exploited for controlled drug release. Multiple types of chemical bonds have already been investigated to meet the drug development requirement that can ease the drug release process. pH-labile covalent bonds with benzoic-imine bond, 1,3,5-triazaadamantane (TAA) group, the hydrazone bond are developed as a proof of principle systems for pH-mediated response systems [67]. Gao et al. have used benzoic-imine bonds to attach α-cyclodextrin directly to mesoporous silica nanoparticles (MSNs) which were partially hydrolyzed in the extracellular tumor space and completely hydrolysed inside endosomes with low pH ~ 5. They have measured the cumulative release of loaded doxorubicin drug in different pH concentrations to confirm the functionality of the system [68].

#### Other stimuli-response systems

Other stimuli have been investigated for controlled release, including heat generated under a magnetic field [49], photo-inducible systems [69], ultrasound inducible systems [70] and electrochemically triggered [71] controlled release of drugs. With current advances in molecular biology and enzyme engineering, there is no limitation to using chemistry methods for surface modification or functionalization of nanoparticles for specificity. Biomolecule incorporation and conjugation methods will assist equally in development of well-controlled drug delivery systems, filling in shortcomings one system presents. Table 1 presents different nanocarriers loaded with drugs that are released to tumor sites based on specific stimuli.

**Table 1:** The stimuli-responsive release of drugs loaded on different nanocarriers

Stimuli	Nanocarriers	Drug	Target	References
pH	Hybrid micelles	Doxorubicin	Breast cancer	[72]
	Mesoporous silica nanoparticles	Doxorubicin	Cervical cancer	[73]
	Dendrimers	Doxorubicin	Breast cancer	[74]
	Coordination polymer mesoporous silica nanoparticles	Topotecan	Cervical carcinoma	[75]
	Gold nanocages	Doxorubicin	Breast cancer	[76]
	Chitosan nanoparticles	Tamoxifen	Breast cancer	[77]
	Polymeric nanoparticles	Cisplatin	Ovarian cancer	[78]
	Titanium dioxide nanoparticles	Daunorubicin	Leukemia	[79]
Redox	Mesoporous silica nanoparticles	Doxorubicin	Glioblastoma	[80]
	Polymeric conjugates	Doxorubicin	Hepatocellular carcinoma	[81]
	Polymeric nanoparticles	Camptothecin, doxorubicin	Breast cancer	[82]
	Magnetic micelles	Doxorubicin	Hepatocarcinoma	[83]
	Chitosan nanoparticles	Methotrexate	Cervical cancer	[84]
	Gold nanoparticles	Doxorubicin, methotrexate, 6-mercaptopurine	Cervical, lung carcinoma	[85]
	Block copolymer nanoparticles	Doxorubicin	Lung cancer	[86]
Magnetic field	Magnetite nanoparticles	Doxorubicin	Multiple myelomas	[87]
	Magnetic nanoparticles	Doxorubicin	Liver cancer	[88]
	Iron oxide nanoparticles	Homocamptothecin	Squamous cell carcinoma	[89]
	PMAM-magnetite nanocrystallites	Cisplatin	Colon adenocarcinoma	[90]
	Superparamagnetic iron oxide nanoparticles	Valrubicin	Prostate cancer	[91]
	Magnetic nanoparticles	Doxorubicin	Cervical cancer	[92]
Light	Mesoporous bamboo charcoal nanoparticles	Doxorubicin	Breast cancer	[93]
	Telluride PEG co-block polymeric nanoparticles	Cisplatin	Breast cancer	[94]
	TiO_2_–iron oxide nanoparticles	Artemisinin	Breast cancer	[95]
	Chitosan-based nanocarrier	Camptothecin	Breast cancer	[96]
Temperature	Liposomes	Tamoxifen, imatinib	Breast cancer	[97]
	β-Cyclodextrin star polymer	Paclitaxel, doxorubicin	Liver cancer	[98]
	Polysaccharide based nanogels	Doxorubicin	Cervical cancer	[99]

Specific nanocarriers, drug and targeted cancer have also been shown in the table

## Influence of physicochemical properties on nanocarriers

The design of highly efficient nanocarriers that meet the requirements for a drug delivery vehicle is an intricate process. A wide range of materials have been used to develop nanocarriers. The primary requirements in precisely engineering these nanomaterials as drug-delivery platforms for sustained release based on their size, shape, composition, surface charge, and biocompatibility, as illustrated in Fig. 1. The physicochemical properties of nanomaterials affect the adhesion to cells, their interaction, and accumulation which leads to therapeutic or toxic effects [23, 100, 101]. Thus, it is fundamental to engineer the nanomaterials to maximize their utility in biomedical applications. The ensuing section discusses major physicochemical properties of nanomaterials and their design considerations for therapeutic and diagnostic applications.

### Size and shape of the nanoparticles

**Fig. 5:**
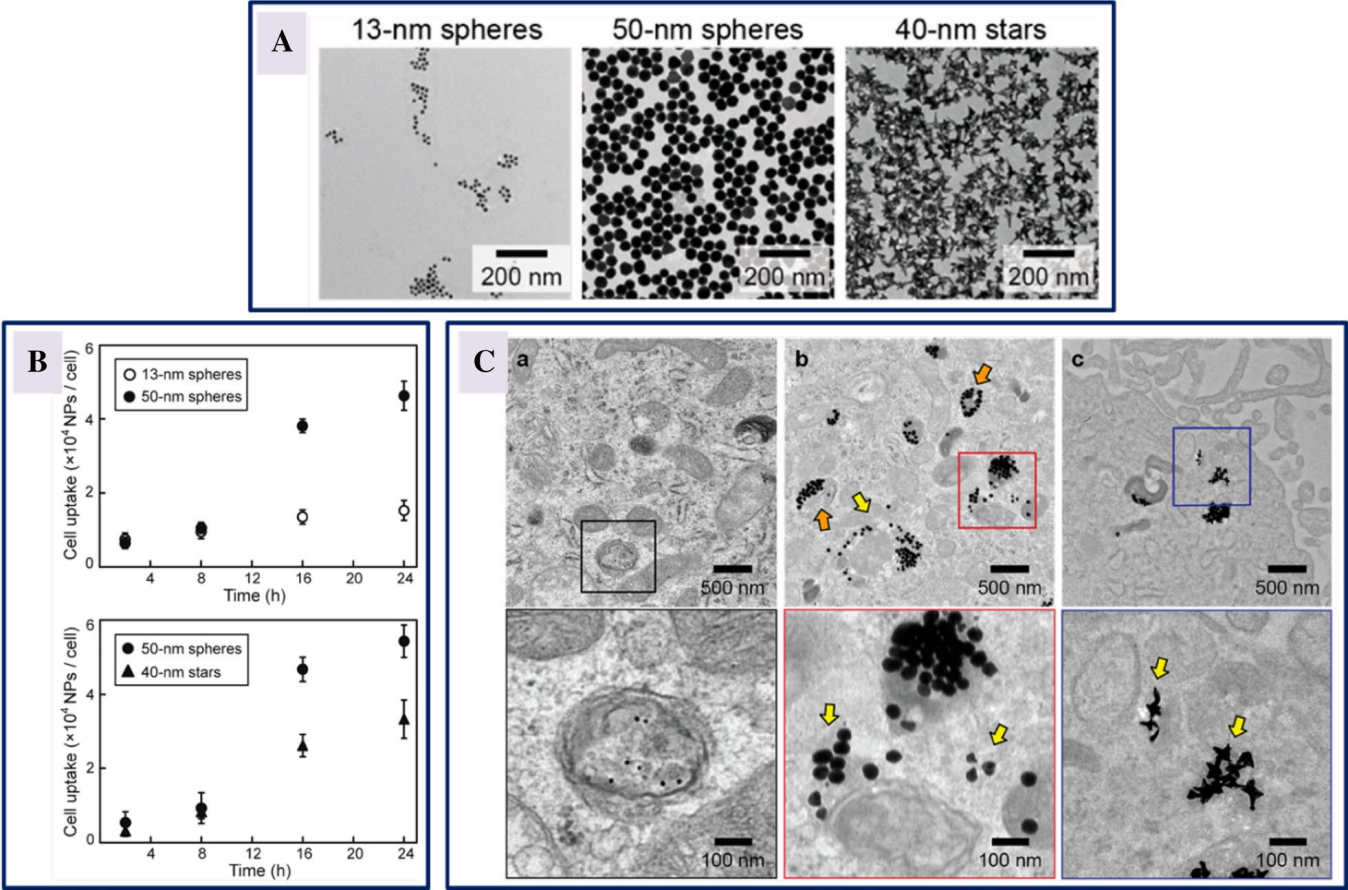
Cellular uptake of gold nanoconstructs by U87 glioblastoma cells. **A** Transmission electron micrographs of Au nanoparticles displaying 13 nm spheres, 50 nm spheres and 40 nm stars; **B** cellular uptake kinetics of Au nanoparticles-siRNA constructs by cells showing size and shape dependent uptake; **C** transmission electron images illustrating the process of cellular uptake after treatment with 0.5 nM of Au nanoparticles-siRNA constructs for 24 h. The vesicle membranes disrupted by the treatment with 50 nm spheres is signified by orange arrows, and the nanoconstructs distributed outside the vesicles is represented by yellow arrows (reproduced with permission from [103])

The size and shape of nanomaterials determine the extent of their tumor accumulation and in vivo distribution. The size of the nanomaterials also influences the uptake of the drug by the cells and interactions with specific tissues for therapeutic purposes. Additionally, the size and shape of the nanomaterials impact the drug loading and release, along with the stability [102]. Recent studies have demonstrated that size and shape of the gold (Au) nanoparticles influence the transfection efficiency of small interfering RNA (siRNA). To ascertain this dependence, three different sizes and two different shapes (13 nm sphere, 50 nm sphere and 40 nm star) of siRNA-conjugated gold nanoconstructs were developed to check the in vitro response of U87 glioblastoma cells targeting the expression of isocitrate dehydrogenase 1. Cellular uptake of larger particles (50 nm spheres and 40 nm stars) was higher when compared to 13 nm spheres, establishing that the size and shape of the nanoconstructs not only influenced the kinetics of cellular uptake but also affected intracellular distribution as depicted in Fig. 5 [103]. In another study, ultrasmall Au nanoparticles of sizes ranging from 2 to 15 nm coated with tiopronin were evaluated for their localization and penetration into breast cancer cells, and it was found that accumulation of smaller nanoparticles was higher in tumor tissues in mice [104]. Similarly, mesoporous silica nanoparticles of different sizes (280, 170, 110, 50 and 30 nm) were examined for the uptake by HeLa cells, revealing the maximum uptake by cells of 50 nm sized mesoporous silica nanoparticles, showing the suitability to be used as carrier vehicles for drug delivery [105].

In addition to the size of the nanomaterials, the shape of the nanomaterials is equally important in drug delivery. Chithrani et al. [106] have studied the effect of the shape of Au nanoparticles (rod and spherical) on cellular uptake and established that the nanoparticles uptake is shape and size dependent, with uptake of spherical nanoparticles efficient compared to their rod-shaped counterparts. Furthermore, silicon-based nanoparticles with quasi-hemispherical, discoidal and cylindrical shapes were used to study the effect of shape-dependent distribution, with discoidal particles distributed to most of the organs tested as compared to other shapes that had less diverse biodistributions [107]. Likewise, Huang et al. have developed different shaped mesoporous silica nanoparticles (sphere, rod, and long rod) functionalized with fluorescein isothiocyanate (FITC) and rhodamine B isothiocyanate (RITC) for imaging and quantification of mesoporous silica nanoparticle uptake. The results designated that long rods are more easily internalized by A375 human melanoma cells, when compared to short rods and spheres shapes [108]. From the above discussion, it can be concluded that nanomaterials for therapeutic applications need to be engineered carefully with respect to their size and shape, because both of them have noteworthy impact on the uptake process of cells, and can potentially induce cellular responses.

### Surface charge of nanoparticles

Like other physicochemical properties, the surface charge of nanomaterials governs their biomedical potency and applicability. The surface charge of the nanoparticles is one of the leading factors to direct the interaction at the nano-bio interface [23]. The cellular entry of nanomaterials depends on surface charge [109]. Additionally, the in vivo biodistribution of nanoparticles suggest that the negatively charged particles accumulate in tumor sites more efficiently [110]. Similarly, the cellular uptake and in vivo fate of micellar nanoparticles have been explored, wherein negatively charged micellar nanoparticles were taken up by tumor cells, and the mechanism of internalization was determined to occur through multiple distinct endocytic pathways including clathrin-mediated endocytosis, caveolae-mediated endocytosis, and macropinocytosis. Likewise, the in vivo distribution of nanoparticles indicated that highly charged nanoparticles were taken up by liver cells. Uptake was less effective with the negatively charged particles, however, indicating the role of negative surface charge on the nanoparticles, which can reduce the undesirable clearance by liver cells [111]. Finally, the surface charge significantly affects the internalization process and the cellular endocytosis mechanism as discussed above [112].

In general, positively charged nanomaterials may internalize efficiently at cell membranes, because of the negative charge on the cell surface [113]. This phenomenon can be further exploited for potential therapeutic purposes, employing nanoparticles as drug or gene delivery carriers. Insightful results have been obtained in the recent past, when cationic liposomes were developed to target the tumors that accumulated in tumor tissues [114, 115]. Moreover, the studies of Villanueva et al. have demonstrated that the internalization of magnetic nanoparticles inside HeLa cells is dependent on the nanoparticle surface charge and incubation time. Specifically, cationic magnetic nanoparticles are retained by the cells for extended period, inducing no cytotoxicity [116]. Additionally, charge switchable nanoparticles have also been developed, and such nanoparticles are reported to change their surface charge in response to external stimuli, with such charge switchable nanoparticles having positive impact toward enhanced cellular uptake [117, 118]. From the discussion above, it is evident that the surface charge of the nanomaterials affects their cellular uptake, and these particles can be efficiently used in cancer treatment based on the cell type and mechanism of endocytosis. Also, it is apparent that the nanomaterials distribution within the cell is strongly governed by their surface charge, which needs to be engineered to avoid undesirable uptake from the normal cells to achieve target specific action without adverse impact on normal cells.

### Surface chemistry of nanoparticles

To develop nanomaterials for specific biomedical applications, surface chemistry design is indispensable. In addition to tailoring the surface corona, engineered nanomaterials reduce their toxicity and enhance their stability [23, 44, 119]. In this context, many studies have demonstrated that cellular interactions of polymer-based nanomaterials are highly influenced by their surface chemistry [120–122]. Recently, PLGA [poly(lactic-*co*-glycolic acid)] based nanomaterials have been developed, demonstrating that suitable surface coating of the nanomaterials provides extended circulation time. This increased circulation time can also lead to higher potency and specific antitumor activity. As an example, drug-coated nanoparticles completely inhibited lung tumor in mice, leading to enhanced survival rate and reduced adverse effect when compared to the free drug [123]. Similarly, PLGA nanoparticles were coated with polyvinyl alcohol (PVA) or vitamin E TPGS to evaluate cellular uptake by Caco-2 cells. The cellular uptake of surface modified PLGA nanoparticles were in the order of vitamin E TPGS-coated PLGA > PVA-coated PLGA > naked PLGA nanoparticles [124]. This pronounced variance from different surface coating suggests that chemical modification of nanoparticles is one of the most effectual means to control and restrain cellular interactions of nanomaterials, and hence their biological consequences.

The surface chemistry of Au nanoparticles and their use in cancer treatment have been extensively studied [125, 126]. The influence of surface coating on the toxicity and cellular uptake of Au nanorods were studied revealing the surface chemistry dependent cellular uptake of Au nanorods covered with poly(diallyldimethylammonium chloride) [PDDAC] [127]. Likewise, PEG capped Au nanoparticles coated with [Pt(1*R*,2*R*-diaminocyclohexane) (H_2_O)_2_]2NO_3_ were taken up, and localized in the lung epithelial and colon cancer cell lines showing more significant effects than the drug alone [128]. Similarly, mesoporous silica nanoparticles coated with different functional groups resulted in different mechanisms of endocytosis by HeLa cells, providing evidence of surface functional group-dependent uptake [129]. Likewise, functionalized carbon nanotubes are extensively used as drug delivery vehicles for delivering small interfering RNA (siRNA), paclitaxel and doxorubicin (DOX) [130–133]. The surface chemistry of the nanomaterials can provide control of the therapeutic effects by reducing the potential undesired side effects. However, surface functionalization needs to be systematically studied before clinical translation. In addition to the above discussion, there are tools that are currently available to shield nanomaterials for targeting cancer cells. Further, antibodies, small proteins, peptides, nucleic acid-based ligands, aptamers, small molecules, and oligosaccharides are used as targeting ligands [134–139]. Nevertheless, it is essential to choose the right type of ligand for improved and efficient targeting of the tumor cells. Since the fate of nanoparticles may be altered due to the surface conjugation of ligands, the nanomaterials further need to be carefully investigated, following their surface decoration to reduce unwanted toxicity effects, and to evaluate their increased specificity and sensitivity post-modification.

In summary, the physicochemical properties such as size, shape, surface charge, and surface chemistry influence the mechanisms of cellular uptake, distribution and therapeutic nature of material. In addition, many other factors have a profound consequence on nanomaterials uptake and distribution in cells. The purity of the nanoparticles, surface to volume ratio, chemical composition, aggregation states, crystal planes, stability, nanoparticle–protein interactions, incubation conditions, cell types, cell treatment, and other factors may also contribute to the cellular uptake and distribution.

## Nanomaterial as drug delivery agents

A wide range of nanotherapeutics, composed of organic and inorganic nanomaterials have been developed with multiple types of drugs or molecules for cancer imaging, detection and treatment. In this section, multiple nanocarriers have been discussed including liposomes, dendrimers, polymeric nanoparticles, and metal nanoparticles.

### Inorganic nanoparticles

This category of nanomaterials forms a significant fraction of current drug delivery systems due to their precise control of size and shape, tuneable physicochemical properties, controlled surface chemistry and diverse multifunctionality. A range of inorganic nanomaterials have been developed in recent past with meticulous properties and employed in biomedical applications especially in cancer treatment and management. Among the inorganic nanomaterials, metal nanoparticles and metal oxides have gained noteworthy consideration due to their exceptional properties and recent progress in the fundamental understanding through the development of innovative techniques. Other major nanomaterials that have noticeable contribution in drug delivery are carbon-based nanostructures and mesoporous silica nanoparticles. Table 2 highlights various inorganic nanocarriers for delivery of anticancer therapeutics. The table illustrates the type of inorganic nanomaterial used as nanocarrier, the explicit drug loaded on the carrier and the cancer cells.

**Table 2:** Overview of various inorganic nanocarriers for delivery of anticancer therapeutics

Nanocarrier	Materials	Drug	Target	Refs.
Metal nanoparticle	Pluronic-b-poly(l-lysine) and gold nanoparticles	Paclitaxel	Human breast cancer (in vitro/in vivo)	[160]
	Folic acid, transferrin and gold nanoparticles	Gemcitabine	Human mammary gland breast adenocarcinoma (in vitro)	[161]
	Apatite stacked gold nanoparticles	Docetaxel	Human liver cancer (in vitro)	[140]
	Chitosan and gold nanoparticles	Doxorubicin	Human breast cancer (in vitro)	[142]
	CTAB and gold nanoparticles	Fluorouracil	Human skin cancer (in vitro/in vivo)	
	Polyethylenimine and silver nanoparticles	Paclitaxel	Human liver carcinoma (in vitro)	[162]
	Silver nanoparticles	Imatinib	Human breast adenocarcinoma (in vitro)	[163]
	PEG and silver nanoparticles	Methotrexate	Human breast cancer (in vitro)	[164]
Metal oxide nanoparticle	PEG and gadolinium oxide nanoparticles	Doxorubicin	Human lung carcinoma, human pancreas ductal adenocarcinoma, human glioblastoma (in vitro)	[165]
	Folic acid, PEG and superparamagnetic iron oxide nanoparticles	Doxorubicin	Human breast cancer (in vitro*/*in vivo)	[166]
	BSA, folic acid and nickel oxide nanoparticles	Doxorubicin	Human cervical epithelial malignant carcinoma (in vitro)	[167]
	PEG and superparamagnetic iron oxide nanoparticles	Doxorubicin	Human colorectal adenocarcinoma (in vitro/in vivo)	[168]
	Zinc oxide nanoparticles	Doxorubicin	Human breast cancer, human colorectal adenocarcinoma (in vitro/in vivo)	[169]
	Superparamagnetic iron oxide nanoparticles	Docetaxel	Human prostate carcinoma (in vitro)	[170]
	PEG, dextran, superparamagnetic iron oxide nanoparticles	Cetuximab	Human squamous carcinoma	[171]
Carbon nanomaterial	PEG and single-walled carbon nanotubes	Cisplatin	Head and neck cancer (in vitro*/*in vivo)	[172]
	PEG, anionic polymer, dimethylmaleic acid and carbon dots	Cisplatin IV	Human ovarian carcinoma (in vitro/in vivo)	[173]
	Chitosan, single walled carbon nanotubes	Doxorubicin	Human cervical epithelial malignant carcinoma (in vitro)	[174]
	Endoglin, iron, single-walled carbon nanotubes	Doxorubicin	Murine breast cancer (in vitro*/*in vivo)	[175]
	Carbon nanoparticles	Methotrexate	Human lung carcinoma (in vitro)	[176]
	Human serum albumin, single-walled carbon nanotubes	Paclitaxel	Human breast cancer (in vitro)	[177]
	Carboxymethyl chitosan, fluorescein isothiocyanate, lactobionic acid, and graphene oxides	Doxorubicin	Human hepatocarcinoma (in vitro)	[178]
	PEG, nanographene oxides	Resveratrol	Mouse mammary carcinoma (in vitro/in vivo)	[179]
	Dendrimer, gadolinium diethylene triamine pentaacetate, prostate stem cell antigen monoclonal antibody, graphene oxides	Doxorubicin	Prostate cancer (in vivo)	[180]
Mesoporous silica nanoparticle	PEG, amino-β-cyclodextrin, folic acid, mesoporous silica nanoparticles	Doxorubicin	Breast cancer (in vivo)	[181]
	Lanthanide doped upconverting nanoparticle, mesoporous silica nanoparticles	Doxorubicin	Murine hepatocellular carcinoma (in vitro*/*in vivo)	[73]
	Bismuth(III) sulphide nanoparticles, mesoporous silica nanoparticles	Doxorubicin	Multidrug-resistant breast cancer (in vitro*/*in vivo)	[182]
	(*S*)-2-(4-isothiocyanatobenzyl)-1,4,7-triazacyclononane-1,4,7-triaceticacid, PEG, Hollow mesoporous silica nanoparticles	Sunitinib	Human glioblastoma (in vitro*/*in vivo)	[183]
	Poly(2-(diethylamino)ethyl methacrylate), Hollow mesoporous silica nanoparticles	Doxorubicin	Human cervical epithelial malignant carcinoma (in vitro)	[184]
	Folic acid, dexamethasone, mesoporous silica nanoparticles	Doxorubicin	Human cervical epithelial malignant carcinoma (in vitro)	[185]
	Glucose, poly(ethylene imine), mesoporous silica nanoparticles	Celastrol	Human cervical epithelial malignant carcinoma, human lung carcinoma (in vitro)	[186]
	Aptamer, mesoporous silica nanoparticles	Doxorubicin	Colon cancer (in vitro)	[187]

The table illustrates the type of inorganic nanomaterial used as nanocarrier, the drug loaded on the carrier and target site

#### Metal nanoparticle and metal oxides

Metal and metal oxide nanoparticles are one of the most useful materials as drug delivery vehicles due to their controllable size and shape, biocompatibility and easy surface functionalization. The noble metal nanostructures, particularly Au nanoparticles, are widely used for delivering drugs [140–142]. Recently, Wan et al. have reported the in vitro anticancer effects of docetaxel conjugated Au doped apatite. Wherein, the material display higher cytotoxicity against human liver cancer cells HepG2, and revealed to have improved bioavailability at the site [140]. Furthermore, Au nanoparticles coated with Pc4, a fluorescent photodynamic therapy (PDT) drug have been developed by functionalizing prostate-specific membrane antigen (PSMA-1) ligand to actively target the disease biomarkers to increase tumor residence time, and internalization by receptor-mediated endocytosis. The efficacy of a theranostics for prostate cancer has also been evaluated through in vitro and in vivo studies [141]. The in vitro studies discovered that the nanoparticle–drug conjugate was more efficient in killing PMSA-expressing cells. This observation provides insights on the active targeting and delivery of Pc4 drug due to the conjugation of Au nanoparticles with PMSA-1 ligand and internalization via clathrin-mediated endocytosis. The in vivo studies have further established that tumor volume reduces post-PDT as demonstrated by the decrease in fluorescence intensity. In one of the recent reports the drug release and stability of pH-sensitive Au nanoparticles loaded with 5-fluorouracil capped with cetyltrimethylammonium bromide (CTAB) was achieved by incorporating into gel and cream bases [142]. The ex vivo permeability of these formulations tested on mice dorsal skin and in vivo anticancer activity were evaluated in A431 tumor-bearing mice. The study has shown the sustained and pH-dependent release, in which the volume of the tumor reduced compared to the untreated control. The outcomes of the study are promising and recommend a topical nanoformulation to enhance drug efficacy against skin cancer. It has been demonstrated that Au nanoparticles decorated with two different anticancer drugs not only prolong the drug circulation time but also enhanced drug targeting and reduced the risk of drug resistance [143].

Similar to Au nanoparticles, silver (Ag) nanoparticles have also been demonstrated to be used as anticancer agents for the treatment of multiple types of cancer [144–147]. Ag nanoparticles have been used to deliver drugs that can elevate the therapeutic indices of the drug [148]. Ag nanoparticles conjugated with phytopharmaceuticals can serve as non-toxic delivery vehicles, contrast agents and photothermal agents for cancer therapy. Biogenic Ag nanoparticles can be employed against prostate and colon cancer. For instance, Ag nanoparticles synthesized using *Indigofera hirsuta* leaf extract and pollen extract of *Phoenix dactylifera* showed dose-dependent cytotoxicity against different cancers [149, 150]. A unique drug delivery system in which Ag nanoparticles coated with a camptothecin-based polymer prodrug was developed for the sustained release of the drug based on pH sensitivity [151]. Another potential strategy for inhibiting tumor metastasis and overcoming drug resistance was developed by co-delivering the drugs with particles featuring different physicochemical properties [152].

Iron oxide nanoparticles (IONPs) have emerged as theranostic nanoparticles providing a means to address the unmet clinical challenges in the treatment of cancer by imaging drug delivery and tumor response [153–157]. Contemporarily, double receptor targeted iron oxide nanoparticles loaded with paclitaxel drug delivery systems have been developed against prostate cancer [158]. The results demonstrated that these iron oxide nanoparticles are effectively internalized by human prostate cancer cell line PC-3. The in vitro magnetic resonance imaging confirmed the enhanced binding and accumulation of iron oxide nanoparticles in PC-3 cells, when compared with normal prostate epithelial cells. Recently, a theranostic nanoparticle to enhance intra-tumoral drug delivery by overcoming drug resistance and providing image-guided drug delivery by reducing the systemic toxicity was developed using iron oxide nanoparticles. In the study, three different targeted nanoparticles and one non-targeted nanoparticle were used to study the uptake and distribution of iron oxide nanoparticles in the PANC02 mouse pancreatic cancer cell line. The study also demonstrated the detection of residual tumors following intraperitoneal therapy signifying the possibility of image-guided surgery to remove drug-resistant tumors [159].

**Fig. 6:**
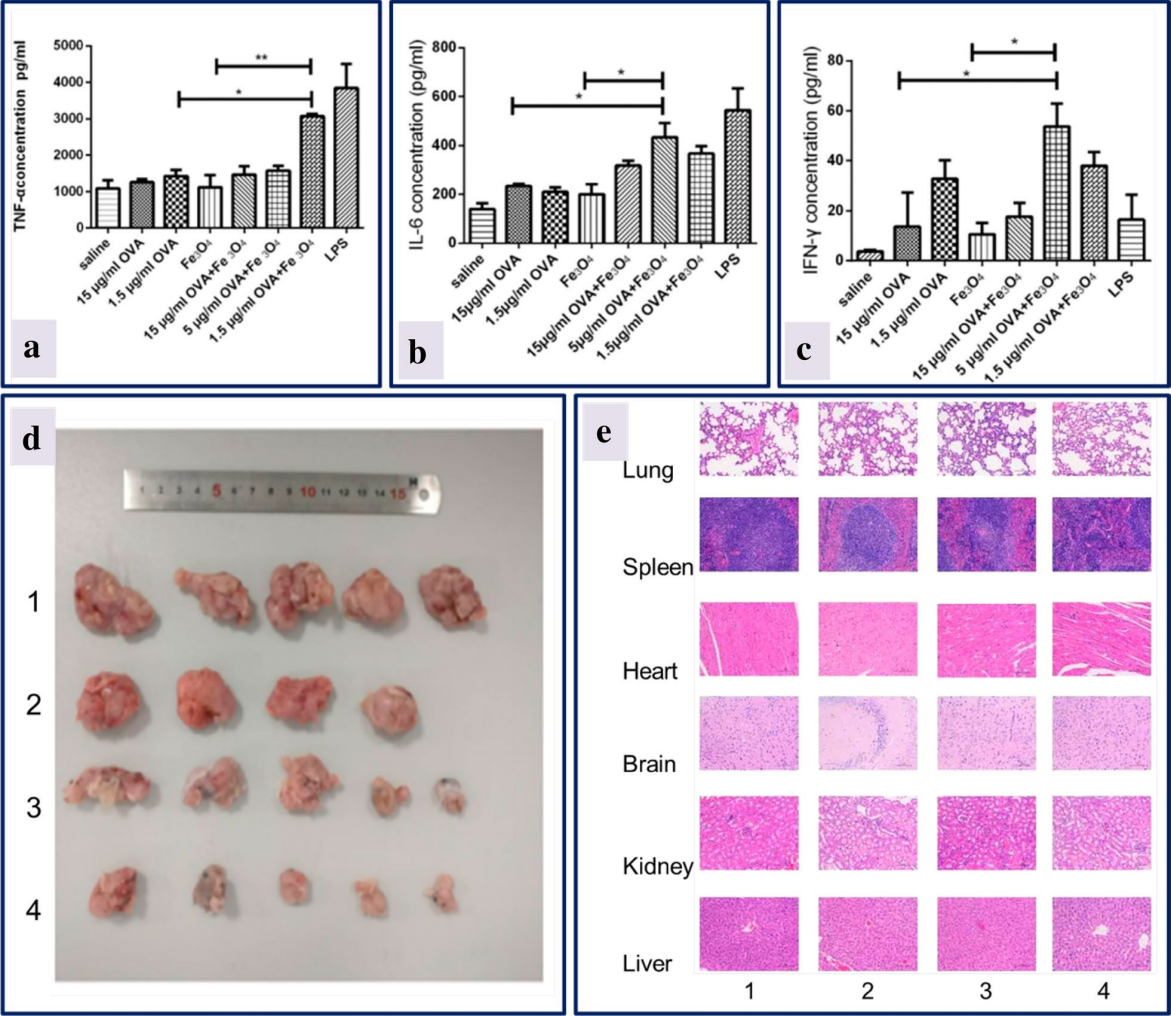
Effect of OVA-iron oxide nanoparticles: macrophages activation with different concentrations of OVA, and production of **a** TNF-α, **b** IL-6, **c** IFN-γ. Saline and LPS served as negative and positive control; **d** size of the tumor measured after 22nd day of mice immunization; **e** histological sections of different organs on 23rd day after immunization of mice with different treatments (1) control, (2) soluble OVA, (3) iron oxide nanoparticles and (4) OVA-iron oxide nanoparticles (reproduced with permission from [188])

In another study, iron oxide nanoparticles were used to deliver OVA, an anticancer vaccine. OVA formulated with iron oxide nanoparticles significantly promoted the activation of immune cells and cytokine production, inducing potent humoral and cellular immune responses. The data indicated that OVA-iron oxide nanoparticles inhibited tumor growth effectively in mice and had good tissue compatibility with organs after intra-tumoral injection as depicted in Fig. 6 [188].

Apart from iron oxide nanoparticles several other metal oxide nanoparticles have been constructed and used for imaging, and drug delivery applications [165, 189–191]. However, their use is often limited due to the accumulation of metal in the body after drug administration causing toxicity. It is recommended that additional studies must be carried out to address the toxicity concerns, since the metal-based nanoparticles are easy to tune with the required properties for efficient loading of drugs and their potential may be excessively high in the field of biology and medicine.

#### Carbon-based materials

Carbon-based nanomaterials have also been extensively studied in imaging, delivery and diagnosis of cancer, due to their attractive characteristics such as high surface area, high drug loading capacity, and easily modifiable surfaces [7, 192–197]. Among the carbon nanomaterials, carbon nanotubes (CNTs) and graphene have been most commonly investigated in cancer therapeutic applications. Carbon nanotubes can assist as drug delivery systems for effective targeting to cancer cells. Recent investigations on multi-walled carbon nanotubes (MWCNTs) for the co-delivery of drugs have revealed that the release of drug at the cancer site, and the uptake by the cells showed the potential for treating multi-drug resistant cancer [198]. Wang et al., developed a multi-walled carbon nanotube platform with improved circulation half-life, and active targeting ability with high drug loading ratio. A pH sensitive nanoplatform can generate heat, following light absorption upon irradiation with near-IR (NIR) light and due to the toxicity of DOX, offering a potential multimodal nanomedicine for efficient cancer treatment [199]. In another report, multi-walled carbon nanotube were decorated with TiO_2_–Au nanocomposite, and the system was observed to be efficient in inducing toxicity to A549 and MCF7 cancer cell lines [200]. Likewise, intracellular drug delivery can be enhanced by utilizing carbon nanotube-based phototherapies. In this context, Levi-Polyachenko et al. have demonstrated increased cell membrane permeability by hyperthermia from multi-walled carbon nanotube, thereby enhancing drug delivery to tumor targets [201]. Similarly, complete tumor eradication has been achieved employing cut-single walled carbon nanotubes coated with BSA-reduced Au nanoparticles, enhancing doxorubicin drug release when combined with phototherapy with an 808 nm laser in a nude mouse model [202]. Alginate and chitosan coated single walled carbon nanotubes loaded with curcumin could target human lung adenocarcinoma (A549) cells, as shown in one recent report [203].

The delivery of PEGylated multi-walled carbon nanotubes conjugated with doxorubicin efficiently released 57% of the drug at lower pH within 24 h, and could inhibit HepG2 cells when compared to free doxorubicin [204]. A recent investigation reported that single walled carbon nanotubes were toxic, and induced death of the organs at higher dosages, whereas multi-walled carbon nanotubes in lower dosages could effectively deliver drug for targeted therapy of abnormal cells in breast cancer [205]. A novel drug delivery system based on carbon nanospheres for delivery of cancer therapeutics has been evaluated for internalization, and possible mechanism of endocytosis and biodistribution in mice [206]. The carbon spheres provided high drug loading capacity along with sustained release of drug under acidic pH, which is the normal tumor microenvironment. In vivo fluorescence imaging revealed the distribution of the drug in organs and these carbon nanospheres exercised antitumor effect in SCID mice bearing oesophageal tumors.

Graphene and its derivatives comprise an important class of materials that is widely used in drug and gene delivery, cell imaging, photothermal cancer therapy and biosensing [207–210]. Recently, a hybrid material based on graphene oxide (GO) coated with β-cyclodextrin (CD) and poly(amido amine) dendrimer (DEN) was used to deliver doxorubicin, camptothecin (CPT) and a photosensitizer (protoporphyrin IX (PpIX)). The drug loading capacity of hybrid material was in the order of camptothecin > protoporphyrin IX > doxorubicin, and displayed enhanced cytotoxicity [211]. A multi-functional graphene oxide based drug delivery system could target cancerous tissues, and exhibit antitumor effect with no systemic toxicity in B16 tumor-bearing mice [212]. To overcome the hypoxia-mediated chemoresistance of oral squamous cell carcinoma (OSCC), platinum loaded, polyethylene glycol-modified graphene quantum dots (GPt) have been utilized. The tested glycol-modified graphene quantum dots exhibited strong inhibitory effect on the tumor growth with minimal systemic drug toxicity in an oral squamous cell carcinoma xenograft mouse tumor model [209].

Multifunctional graphene smart nanomaterials have been developed for drug delivery and cellular imaging in cancer treatment [210, 213]. Recently, nanographene oxide complexed with upconverting nanoparticles were used for tumor imaging and photothermal therapy, signifying the potential of multifunctional graphene for clinical antitumor treatments [213]. The combination of chemotherapy with photothermal therapy has proved to be efficient when magnetic graphene oxide modified with PEG and cetuximab was used against CT-26 murine colorectal cells [214]. The active targeting was achieved using cetuximab, an epidermal growth factor receptor (EGFR) monoclonal antibody, since epidermal growth factor receptor is highly expressed on the tumor surface of colorectal cancer cells. The pH dependent release studies indicated the drug release was greater at pH 5.5 than pH 7.4 and could effectively target epidermal growth factor receptor-expressing CT-26 murine colorectal cells. The in vivo antitumor studies suggested that the tumor volume drastically reduced in mice in the presence of magnetic nanocarrier, magnet and laser. There was 29-fold increase in therapeutic efficacy of the nanocarrier during the combination therapy when compared to control.

Liu et al., developed graphene oxide modified with chitosan followed by conjugation with hyaluronic acid and an anti-cancer drug SNX-2112. The graphene oxide based carrier was found to be effective in inhibiting and killing A549 cells, and displayed lesser toxicity against normal human bronchial epithelial cells [215]. Similarly, graphene oxide with galactosylated chitosan with doxorubicin have been developed for the treatment of cancer. These nanocarriers were stable and their release was reported to be pH responsive. The in vivo antitumor effect of galactosylated graphene oxide was better than the chitosan graphene oxide, which was demonstrated by tumor weight and volume [216]. Clearly, carbon-based nanomaterials have led to the improvement in cancer therapy due to their unique properties. All these observations are motivating and may change the face of cancer treatment and management.

#### Mesoporous silica nanomaterials

Mesoporous silica nanomaterials (MSNs) have emerged as another class of drug delivery carriers, due to their surface properties such as large surface area, uniform porosity, stability, low toxicity and narrow size distribution [217]. The designing of multifunctional delivery platforms using mesoporous silica nanomaterials with different characteristics is possible because of facile modification of their surface. These features have led many researchers to load cargos on to mesoporous silica nanomaterials for transporting them to the tumor tissues [218–220]. Many anticancer drug applications are limited due to its solubility, stability, and bioavailability of the drug. In this context, Li et al., have developed novel nanocarrier systems for tumor targeting and precise release of curcumin. These surface modifiable mesoporous silica nanomaterials have been exploited to deliver curcumin to breast cancer cell lines that were loaded with hyaluronan or polyethyleneimine-folic acid and were tested on mouse xenograft model [221]. The folic acid modified mesoporous silica nanomaterials showed an enhanced cellular uptake than hyaluronan mesoporous silica nanomaterials and both nanoformulations had better cellular uptake when compared with that of a non-targeted nanocarrier. These nanoformulations showed better biocompatibility with low toxicity and inhibited tumor growth to a greater extent than curcumin alone. In a related study, to treat the multidrug resistant cancer cells with elevated Bcl-2 levels, Xu et al. [222] have developed macroporous silica nanoparticles with a peptide loading efficiency of 40%, which upon administration induced apoptosis. Often in the breast cancer cells, Mucin 1 (MUC1), a cell surface protein, will be overexpressed. In vivo studies of MUC1 aptamer-capped mesoporous silica nanomaterials on MDA-MB-231 tumor-bearing Balb/c mice were found to effectively target breast cancer cells and induce a dramatic reduction in cell viability [223].

Additionally, mesoporous silica nanomaterials can release cargo in response to stimuli. These smart nanosystems trigger the release of the drug trapped in the pores to the target sites in the presence of either endogenous or exogenous stimuli, with control on the administered dose. The pH responsive release of the drug is widely employed, since the tumor microenvironment will be slightly more acidic than the normal tissues. Liu et al. [224], utilized hollow mesoporous silica nanomaterials to release doxorubicin to HeLa cells in an acidic environment exhibiting anticancer effect with good biocompatibility. Additionally, mesoporous silica nanomaterials for the CD44-targeting pH responsive smart drug delivery system were developed by hyaluronic acid end-capping and loaded with doxorubicin. The fabricated nanoparticles enhanced cellular uptake via CD44 receptor-mediated endocytosis by HeLa cells. The cytotoxicity of doxorubicin-loaded mesoporous silica nanomaterials toward cancer cells overexpressing CD44 receptor was enhanced with IC_50_ of 0.56 μg/mL whereas; the normal cells showed lower cytotoxicity with the IC_50_ of 1.03 μg/mL [225]. Likewise, external stimuli mediated treatment of cancer with mesoporous silica nanomaterials is seen as suitable drug delivery candidates since the external stimuli will be independent of complicated tumor physiological microenvironment. Recently, core–shell nanoparticles were also developed with a magnetic core and mesoporous silica nanomaterials shell to effectively deliver epirubicin. There was a 27% increase in the cellular uptake of cells treated with magnetic mesoporous silica nanomaterials with epirubicin in the presence of external magnetic field when compared to free epirubicin [226].

Multifunctional mesoporous silica nanomaterials have been employed to provide a synergistic blend of different assemblies into nanoplatforms with enhanced antitumor activity and less cytotoxicity to normal cells. These particles can selectively target human osteosarcoma cells and are capable of pH-responsive antitumor drug delivery. The nanosystems exhibited higher internalization degree into human osteosarcoma cells and induced almost 100% osteosarcoma cell death with a low doxorubicin loading of 2.5 µg/mL. The cytotoxicity of nanoplatform was eightfold higher than that of the free drug [227]. It is evident that mesoporous silica nanomaterials are one of the promising nanocarriers for efficient delivery of cancer therapeutics due to their useful properties. The possibility of using mesoporous silica nanomaterials as potential nanocarriers has driven interest in many biomedical applications. However, more in-depth studies are required to understand the pharmacokinetic and pharmacodynamic properties of these systems before clinical translation of mesoporous silica-based nanomaterials.

### Organic nanomaterials

Organic nanomaterials are promising candidates for the development of drug delivery systems. Significant properties of any nanomaterial used in biomedical delivery are its biocompatibility and biodegradability [228], with the discharged carrier degraded into nontoxic components and cleared through the circulation. These attractive properties along with low toxicity have enabled the nanomedicine research community to use organic nanomaterials as drug delivery vehicles to target specific tissues and controlled release of the drug molecules. To date, many types of organic nanocarriers have been developed such as liposomes, polymeric nanoparticles, dendrimers and micelles. A summary of different organic nanomaterials used as drug delivery carrier for anticancer drugs and the targets is shown in Table 3.

**Table 3:** Summary of different organic nanomaterials used as drug delivery carrier for anticancer drugs

Nanocarrier	Materials	Drug	Target	References
Liposomes	Hyaluronic acid–ceramide and egg phosphatidylcholine	Doxorubicin	Human breast cancer (in vitro/in vivo)	[247]
	DSPE-PEG2000-Pen, DSPE-PEG2000-Tf	5-Fluorouracil	Human glioblastoma (in vitro)	[248]
	DPPC, MPPC	Tamoxifen, imatinib	Human breast cancer (in vitro)	[97]
	DPPC, cholesterol, DSPE-PEG-FA	Celastrol and irinotecan	Human Breast cancer (in vitro/in vivo)	[236]
	DSPE-PEG2000-NHS, pHCT74 peptide	Doxorubicin	Human prostate cancer (in vitro/in vivo)	[249]
	CHEMS, DOPE, DSPE-PEG2000	Tariquidar and doxorubicin	Human ovarian cancer (in vitro)	[241]
	Egg phosphatidylcholine, DOPE, CHEMS, DSPE-PEG2000	Resveratrol	Human glioblastoma (in vitro)	[237]
	PC, DSPE-PEG2000	Doxorubicin and celecoxib	Human skin cancer (in vitro)	[250]
Polymeric nanoparticles	PLGA [poly(lactic-*co*-glycolic acid)], PVA [poly(vinyl alcohol)]	Abiraterone acetate and docetaxel	Human prostate cancer (in vitro)	[251]
	MPEG-PVA [poly(vinyl alcohol)]	Verapamil and doxorubicin	Human ovarian cancer (in vitro)	[252]
	PLGA [poly(lactic-*co*-glycolic acid)], PVA [poly(vinyl alcohol)]	Resveratrol	Human prostate cancer (in vitro)	[253]
	PLA	Calcitriol	Human breast cancer (in vitro)	[254]
	TPGS-b-PCL, Pluronic P123	Sorafenib	Human liver carcinoma (in vitro/in vivo)	[255]
	PLGA [poly(lactic-*co*-glycolic acid)], PEG, chitosan	Curcumin	Human pancreatic cancer (in vitro)	[256]
	PLGA [poly(lactic-*co*-glycolic acid)], DSPE-PEG	Docetaxel	Human tongue carcinoma (in vitro)	[257]
Dendrimers	PAMAM, octa-arginine, PEG	Paclitaxel	Human cervical carcinoma (in vitro)	[258]
	PAMAM, *N*-acetylgalactosamine ligand, PEG	Doxorubicin	Hepatocellular carcinoma (in vivo)	[259]
	PAMAM, lactobionic acid	Sorafenib	Human liver cancer (in vitro)	[260]
	PAMAM, folic acid	Baicalin	Human cervical cancer (in vitro)	[261]
	PAMAM, PEG, AS1411-aptamer	Camptothecin	Human colon adenocarcinoma (in vitro/in vivo)	[262]
	PAMAM, OEG	Methotrexate	Human breast cancer (in vitro/in vivo)	[263]
	h-PAMAM, PEG-SC	Doxorubicin	Human gastric cancer (in vitro/in vivo)	[264]

The table also shows the type of nanomaterial employed as nanocarrier and target site

#### Liposomes

**Fig. 7:**
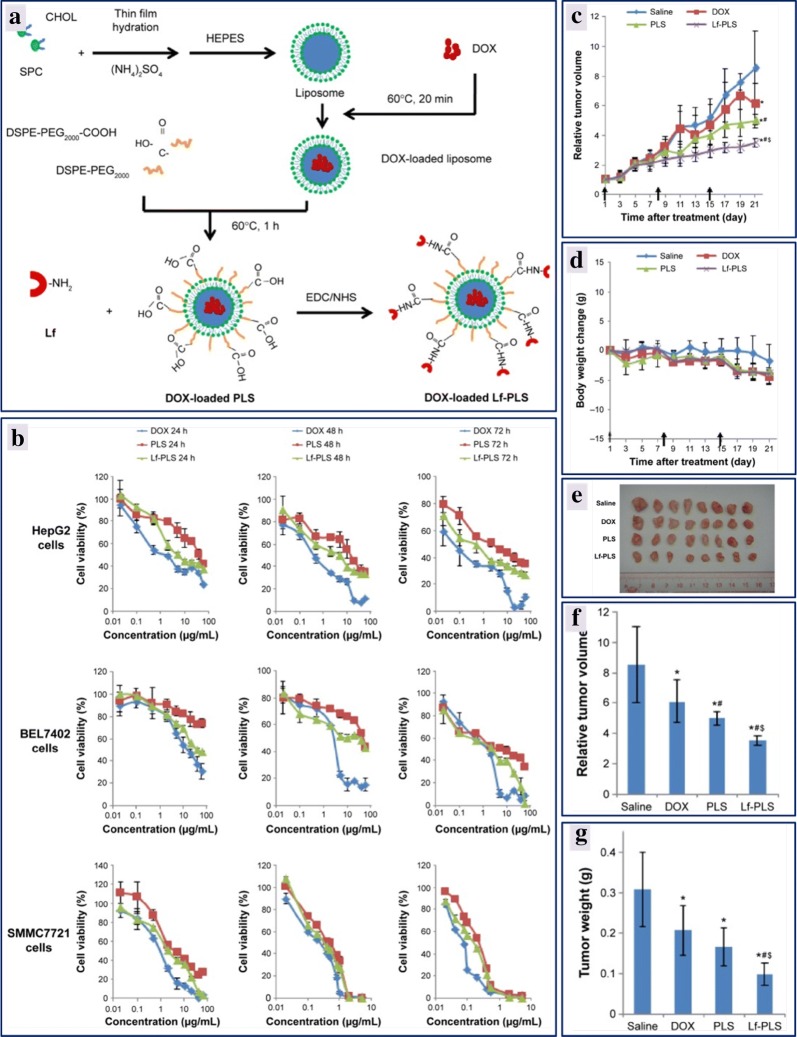
Scheme representing the formulation of doxorubicin loaded PEGylated liposome, and doxorubicin loaded lactoferrin modified PEGylated liposome (**a**); effect of cell viability of free DOX and the liposomal formulations evaluated by MTT assay in HepG2, BEL7402, and SMMC7721 cells at different time intervals (**b**); relative tumor volume of various liposomal formulations injected to tumor-bearing mice through tail veins every 7 days at a dose of 5 mg/kg DOX (**c**); change in the body weight of tumor-bearing mice after each treatment (**d**); image of tumors excised on 21st day from each treatment group (**e**); relative tumor volume at the time of sacrifice from each treatment group (**f**); tumor weight at the time of sacrifice from each treatment group (**g**) (reproduced with permission from [235])

The use of a nanoparticles for medicine was first described in 1965, with liposomes as the first ones to be used [229]. Liposomes are spherical vesicles composed of a lipid bilayer of either synthetic or natural phospholipids surrounding an internal aqueous phase. The structure of liposomes can be engineered to encapsulate the hydrophobic or hydrophilic drugs, or other small molecules, in the lipid bilayer or aqueous core, respectively [230]. Liposome-based drug carrier systems have been developed to prolong the circulation time of the drugs and reduce toxicity to healthy tissues around. Correspondingly, these vehicles offer several other advantages including biocompatibility, self-assembly, and high drug cargo loading [231]. Due to the morphological similarity with cellular membranes and ability to integrate with various substances, liposomes serve as an ideal drug-carrier systems. Over the past 20 years, commendable progress has been made in biomedical applications of liposomes improving the therapeutic index of the encapsulated drugs. There are different classes of liposomes used as drug delivery platforms for enhancing the efficacy of cancer therapeutics [232]. Liposomes can be conjugated with poly(ethylene glycol) (PEG), targeting ligands and/or antibodies, polysaccharides on the external surface to enhance solubility, to increase the hydrophilicity and to provide passive and active targeting functions, in due course attaining high drug efficiency with low toxicity [233]. By exploiting the extended circulation property of PEGylated liposomes and biocompatibility, biodegradability and hydrophilicity of polysialic acid, a negatively charged polysaccharides drug delivery systems developed that has been used to prolong the circulation time of the liposomes, increasing the ability of epirubicin to reach the tumor sites. These antitumor studies revealed that modified liposomes had lower systemic toxicity and prolonged the survival time of the treated mice by suppressing the tumor growth more strongly [234]. Likewise, doxorubicin-loaded modified PEGylated liposomes were developed for targeted delivery of drug to hepatocellular carcinoma. Soybean phosphatidylcholine/cholesterol was used in the molar ratio of 3:2 to prepare liposomes by thin film hydration method, and doxorubicin was remotely loaded into the liposomes via the ammonium gradient method. Later liposomes were PEGylated (PLS) by a PEG-lipid post-insertion technique followed by covalent coupling with lactoferrin (Lf) to the surface of liposomes as illustrated in Fig. 7a. These liposomal formulations exhibited negative zeta potential values and an in vitro release study demonstrated that the liposomal formulations displayed good stability, and an extended circulation time required to avoid drug clearance before arrival at the target cells. The in vitro cytotoxicity studies revealed that doxorubicin formulations had increased antiproliferative effect and was time and dose-dependent as depicted in Fig. 7b. Doxorubicin-loaded lactoferrin-PLS displayed stronger inhibitory effects in ASGPR-positive HCC cells than with unmodified PEGylated liposomes. The in vivo antitumor studies were conducted on male BALB/C nude mice bearing a HepG2 tumor model. Interestingly, there was a significant tumor growth inhibition in the treatment group with doxorubicin-loaded lactoferrin-PLS of HepG2 tumors when compared to only doxorubicin-loaded PLS and free doxorubicin, with no significant change in the body weight observed as shown in Fig. 7c–g. This important study signposts the strategy of modifying the surface of liposomes for effective delivery of anticancer drugs to treat hepatocellular carcinoma [235]. Similarly, the PEGylated liposomes have been used in delivering celastrol, irinotecan, resveratrol in the treatment of breast cancer and glioblastoma [236, 237].

Approaches for co-delivery of different chemotherapeutics have been developed as a useful method for the treatment of cancer. Combination therapy has been demonstrated to be effective and has substantial evidence showing that synergistic effects that are superior to the totality of the therapeutic consequences of the individual drug [238–240]. Several strategies have also been developed to accomplish liposomal codelivery of chemotherapeutic agents. These liposome-based combinational formulations have significant popularity due to augmented anticancer effects, antiproliferative activity, apoptosis, and cytotoxicity while diminishing the systemic toxicity. Besides, liposomal co-delivery of chemotherapeutic agents can minimize cancer cell drug resistance and make them more sensitive to individual drugs. In a recent study, co-delivery of two chemotherapeutic agents (tamoxifen and imatinib mesylate) using a liposome carrier system was developed to treat breast cancer. Tamoxifen and imatinib mesylate were released in controlled manner from the temperature sensitive liposomes prepared using a combination of phospholipids with a transition temperature near to 39 °C. The dual drug-loaded thermo-sensitive liposomes exhibited significantly larger release rate of both the drugs at 40 °C and displayed synergistic inhibition of breast cancer cell proliferation. The findings highlighted the development of a thermo-responsive liposomal drug delivery system for combinational breast cancer treatment [97]. Similarly, pH sensitive liposomes have also proved to be effective in increasing the drug accumulation in resistant tumor cells and are potent drug carriers that can overcome multidrug resistance. Xia et al., constructed a pH sensitive liposome formulation by loading tariquidar (TQR) and doxorubicin to overcome multidrug resistance of drug-resistant ovarian cancer cells. The tariquidar and doxorubicin-loaded pH sensitive liposome formulation exhibited outstanding tumour inhibition against the tested cells by increasing the accumulation of doxorubicin in cells, allowing them to enter specifically into the nuclei [241]. Additionally, a newer generation of liposomes are emerging, focusing on redox sensitive liposomes, magnetic liposomes, enzyme sensitive liposomes and multifunctional smart liposomes [242–245].

The above discussion signifies the importance of liposomes in drug delivery systems for the treatment of cancer. These nanocarriers help overcome the unwanted side effects in normal tissues and increase circulation time, bioavailability, and accumulation of drug at target-site by reducing toxicity and protect the chemotherapeutic agents from the surrounding environment. In spite of widespread research and the preclinical development of liposomal formulations from several decades, only a few liposomal drug formulations have been approved by the FDA for clinical use [246]. Formulations have been approved for the treatment of Kaposi’s sarcoma, acute lymphoblastic leukemia, pancreatic cancer, ovarian cancer, multiple myeloma and metastatic breast cancer including Doxil^®^, Myocet^®^, DaunoXome^®^, DepoCyte^®^, Lipoplatin^®^. This major setback has led to the development of ligand-directed liposomes for active targeting and treatment of different types of cancer.

#### Polymeric nanoparticles

Polymeric nanoparticles are colloidal nanoparticles wherein therapeutic molecules will be encapsulated or adsorbed or conjugated in the polymer matrix. These nanoparticles can be synthesized using synthetic and natural polymers, and have been extensively used in drug delivery applications [265, 266]. These nanoparticles can be customized for various biomedical applications due to their unique characteristics such as drug solubility, stability, and preferential accumulation [267]. Polymeric nanoparticles serve as a versatile platform to deliver drugs due to their different chemical composition, charge and physical structure. Moreover, they have gained commercial importance because of their tunable drug release kinetics. Drugs can be efficiently delivered using polymeric nanoparticles by active or passive targeting the cancer cells. Tumor-specific targeting at the surface of the cancer cells has also been explored to eradicate tumor cells. Various ligands such as antibodies, proteins, peptides, aptamers and small molecules have been used to target specific cells [268]. The targeting of cells by nanoparticles results in highly specific delivery of cargos, resulting in high concentrations of the therapeutic within the cell. Several studies have demonstrated enhanced antitumor activity with targeting moieties. Surface modified polylactic acid (PLA) nanoparticles have been reported and employed for delivery of docetaxel (DTX) as a targeted drug delivery system for the treatment of liver cancer. Docetaxel-loaded galactosamine combined with polydopamine-modified nanoparticles synthesized from d-a-tocopherol polyethylene glycol 1000 succinate-poly(lactide) (Gal-pD-TPGS-PLA/NPs) were found to inhibit the growth of HepG2 cells more effectively than TPGS-PLA/NPs, pD-TPGS-PLA/NPs, and a clinically available docetaxel formulation (Taxotere^®^). The in vivo transplantable liver tumor bearing BALB/c nude mice treated with docetaxel loaded Gal-pD-TPGS-PLA/NPs exhibited noticeable tumor growth inhibition when compared to other nanoformulations and free Taxotere^®^. The authors have suggested that the antitumor effect of the surface modified docetaxel loaded polylactic acid nanoparticles resulted from the targeted delivery to HepG2 cells [269]. In another study, resveratrol encapsulated PLGA [poly(lactic-co-glycolic acid)] nanoparticles have been constructed for prostate cancer therapy. These nanoparticles exhibited a significant decrease in cell viability and greater cytotoxicity toward LNCaP cells when compared to free resveratrol. The cytotoxicity assay demonstrated that resveratrol conjugated poly(lactic-co-glycolic acid) nanoparticles had two-fold lower IC_50_ and IC_90_ values in comparison to only resveratrol [253].

The combination of multiple drugs has been established to be more effective than single drug treatment. It is anticipated that multiple drugs when delivered simultaneously to a cancer cell will exhibit a synergistic effect, when administered in an optimized ratio. Due to the advancements in nanomedicine, several nanoparticle formulations have been developed for co-delivery of cancer chemotherapeutics [270, 271]. Zhang et al., designed pH sensitive TPGS-PAE nanoparticles, polymeric nanoparticles, wherein doxorubicin and curcumin were co-loaded by self-assembly. The nanoformulation exhibited a high rate of apoptosis against human liver cells and stronger anti-angiogenic effects together with inhibition of proliferation, migration, invasion, and tube formation [272].

**Fig. 8:**
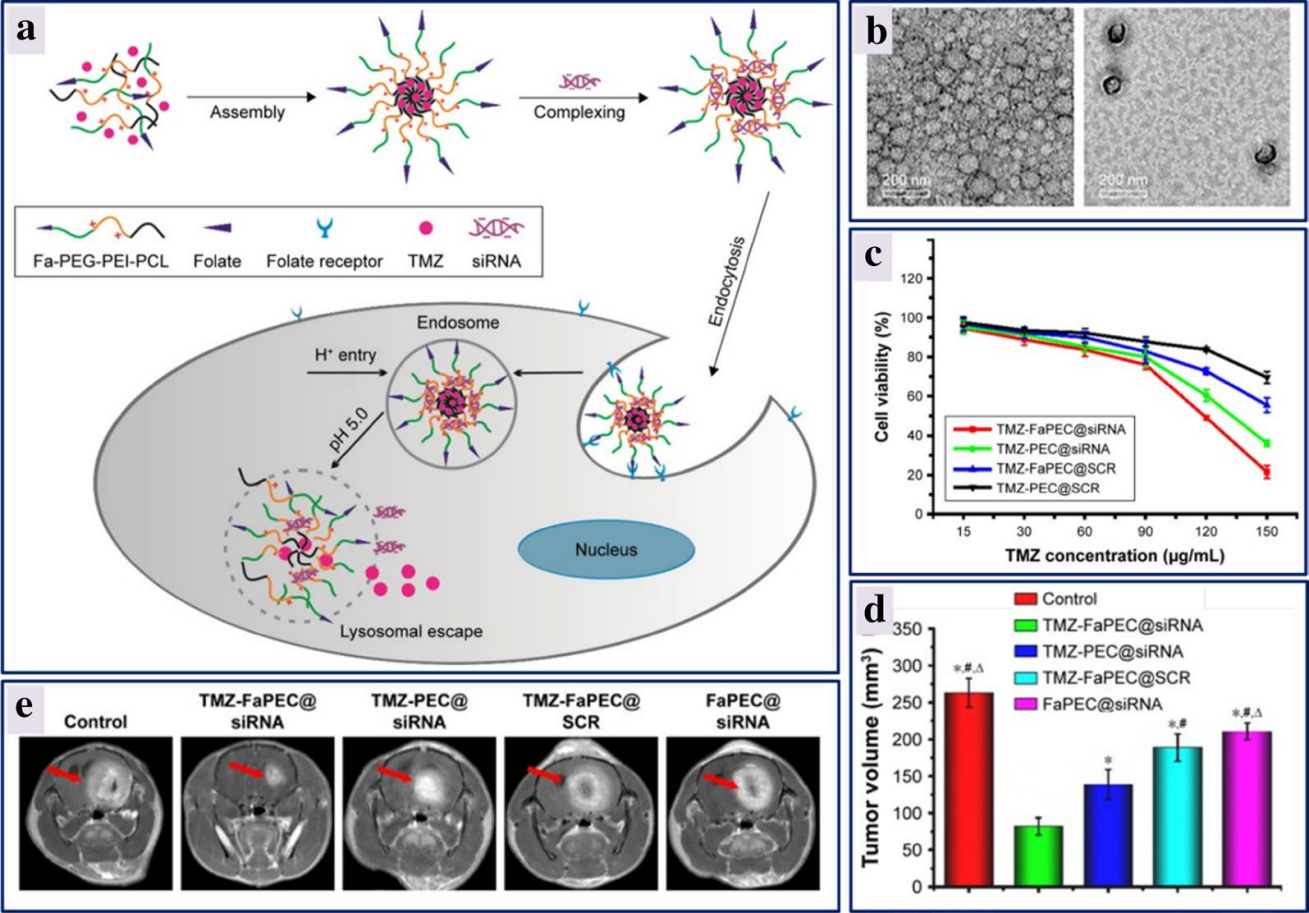
Illustration of TMZ (temozolomide) and siRNA conjugated preparation of folic acid decorated Fa-PEG-PEI-PCL and release of antitumor therapeutics inside the cancer cells (**a**); TEM images showing TMZ-conjugated, folic acid-decorated PEC micelle (left) and TMZ and siRNA-conjugated, folic acid-decorated PEC micelle (right) at pH 7.4. All samples were stained with 0.5% uranyl acetate for 1 min. Scale bar: 200 nm (**b**); in vitro cytotoxicity effect of different nanocomplexes on C6 cells evaluated by CCK8 assay at various TMZ concentrations. Cells were incubated for 48 h and BCL-2 siRNA concentration used is 20 nM (**c**); Mean tumor volume determined using magnetic resonance imaging measured after 25 days of the first injection. *P < 0.05 vs TMZ-FaPEC@siRNA; ^#^*P *< 0.05 vs TMZ-PEC@siRNA; ^Δ^*P *< 0.05 vs TMZ-FaPEC@SCR (**d**); visualization of tumor growth inhibition in male Sprague–Dawley rats implanted with C6 cells after treatment with different formulations (red arrow indicates the tumor) (**e**). Fa, folate; PCL, poly(ε-caprolactone); PEG, poly(ethylene glycol); PEI, poly(ethylenimine); TMZ, temozolomide (reproduced with permission from [273])

Another polymeric nanoparticle platform that is gaining significant attention as drug delivery systems is polymer micelle nanoparticles. Recently, Peng et al. prepared a polymeric micelle by incorporating temozolomide (TMZ) and anti-BCL-2 siRNA based on tri-block copolymer conjugated with folic acid as outlined in Fig. 8a for delivering temozolomide and siRNA to overcome the drawbacks of acquired resistance of glioma cells and restriction of blood–brain-barrier (BBB) for drug delivery. The nanocomplexes were spherical in shape, which was confirmed by transmission electron microscopy analysis as shown in Fig. 8b. Further, as illustrated in Fig. 8c, the cell viability of various formulations was investigated on a rat C6 glioma cell line at different temozolomide concentrations. Temozolomide-FaPEC@siRNA exhibited higher cytotoxicity than both temozolomide-FaPEC and temozolomide-PEC, whereas C6 cells incubated with FaPEC@SCR and PEC@SCR exhibited viabilities over 90% even at a very high 100 µg/mL polymer concentration, indicating low cytotoxicity of carrier, a vital characteristic for in vivo application. In vivo, pharmacokinetic studies have also been conducted in the study to reveal the variation in the glioma growth in rat brain for different complexes after 25 days of the first injection. The tumor volume as depicted in Fig. 8d, e was determined by magnetic resonance imaging and showed that temozolomide and siRNA conjugated nanocomplex had a volume of 82 ± 11 mm^3^ which is much less than the volume resulting with the other treatments. However, the siRNA or temozolomide treatment mediated by the folate-targeted nanocarrier was able to prevent glioma growth, the combination therapy was more effective than the individual treatment [273]. These in vitro and in vivo studies confirmed the effectiveness of combination therapy using temozolomide and siRNA for treatment of glioma and provided understanding on the folate targeted co-delivery of cancer therapeutics. Apart from folate-mediated targeting, aptamer-functionalized PEG-PLGA nanoparticles have also been constructed for anti-glioma drug delivery by active targeting the tumor. PEG-PLGA nanoparticles were conjugated with AS1411, a DNA aptamer, that binds to a protein highly expressed in the plasma membrane of cancer and the endothelial cells of angiogenic blood vessels. The designed nanoformulation was spherical in shape with 156 ± 54 nm size and a negative zeta potential exhibiting increased cytotoxicity in C6 glioma cells. The targeted nanosystem established higher tumor inhibition and prolonged the survival time of rats bearing intracranial C6 glioma, when compared to paclitaxel conjugated nanoparticles and a commercial drug Taxol^®^ [274].

Therefore, polymeric nanoparticles can be effectively used to deliver cancer therapeutics by active and passive targeting. Also, these platforms can provide competent drug delivery systems responsive to various stimuli to enhance the therapeutic efficacy and reduce the side effects of loaded drugs. Polymeric nanoparticles are efficient in enhancing therapeutic and diagnostic effects over conventional medicines. Several polymer-based therapeutics are currently in the market or undergoing a clinical evaluation to treat cancer. Such clinical trials are projected to intensify the use of polymeric drug delivery systems in the near future.

#### Dendrimers

Dendrimers are multi-branched molecules with functional groups on the surface with an inner core. These structures can be produced by using macromolecules such as polyamide amine (PAMAM), polypropyleneimine and poly(aryl ether). The most striking properties of dendrimers such as branches, distinct molecular weight and globular assembly with meticulous surface functionality, and multivalency, can be exploited to be used as carriers for drug delivery [275]. In addition to functional groups on their branches, they are suitable for loading and binding diverse hydrophilic and hydrophobic drugs. These dendritic systems have been used to deliver anticancer drugs wherein the drugs are encapsulated/conjugated with dendrimers. These nanocarriers have demonstrated to decrease non-specific toxicities, improve drug delivery profiles, enhance drug stability and bioavailability, targeted drug delivery.

The solubility, biodistribution and resistance of anticancer drugs together form a significant hurdle in improving the pharmacodynamic profile for the treatment of cancer. To overcome these drawbacks, a polyamide amine dendrimer conjugated with paclitaxel and docosahexaenoic acid (DHA) was developed to enhance the anticancer activity by increasing its efficacy and reducing toxicity. The in vitro studies indicated that the nanocarrier developed with docosahexaenoic acid, polyamide amine and conjugated with PTX had a better anticancer activity toward upper gastrointestinal cancer cells when compared to polyamide amine conjugated with PTX [276]. Likewise, half-generation polyamide amine dendrimers reduce cytotoxicity due to the presence of negatively charged carboxylic or cyano groups on their surface. Several researchers have demonstrated that half-generation dendrimers exhibit lower toxicity than the full generation of polyamide amine [277–279]. Likewise, Thanh et al., generated Heparin-functionalized monomethoxy PEG-polyamide amine dendrimer (HEP-mPEG) with effective encapsulation of DOX. G4.0-polyamide amine-HEP-mPEG revealed precise release of doxorubicin and had prolonged retention compared to pristine doxorubicin in both Hela and fibroblast NIH3T3 cancer cells. The results demonstrated that the high drug loading capacity and less systemic toxicity of G4.0 polyamide amine-HEP-mPEG/DOX could serve as a suitable drug delivery system [280].

The most effective approach of delivering anticancer drugs is by conjugation of ligands that specifically recognize and binds to the receptors on the tumor cells. In this context, Chittasupho et al., have developed CXCR4 targeted dendrimer for breast cancer therapy. Linear type of FC131 (LFC131) ligand conjugated, doxorubicin encapsulated polyamide amine dendrimer was developed using polyamide amine dendrimer generation 4.0 (D4). The cytotoxicity of the dendrimer encapsulated doxorubicin and LFC131-DOX-D4 to BT-549-Luc cells was evaluated and the IC_50_ value of LFC131-DOXD4 was 2.8 fold of DOX-D4 against BT-549-Luc cells and it was 6.8 fold of DOX-D4 against T47D cells after 24 h of incubation, indicating that the ligand conjugated doxorubicin encapsulated dendrimer can enhance the cytotoxicity of the drug against the cancer cell lines [281]. Likewise, Öztürk et al., developed a PEF modified dendrimer-based drug delivery system targeting Flt-1 (a receptor for vascular endothelial growth factors (VEGF)) receptor to improve the therapeutic efficacy of gemcitabine in pancreatic cancer. The CFPAC-1 pancreatic adenocarcinoma cell viability decreased, indicating a PEGc polyamide amine-PEG dendrimers anti-cancer effect. Conjugation with anti-Flt-1 antibody improved the accumulation of PEGc polyamide amine-PEG dendrimers into the pancreatic tumors [282]. Stimuli responsive dendrimers enhance therapeutic efficiency and diminish the side effects. These are responsive to pH, temperature, enzyme, light, the concentration of glutathione [283].

From the above discussion, it is evident that dendrimers are nanoplatforms which can be tuned for therapeutic applications, and show great promise in the treatment of various cancers. The challenge of bench-to-bedside translation of dendrimers, however, remains a significant challenge.

## Challenges in nano drug delivery

**Fig. 9:**
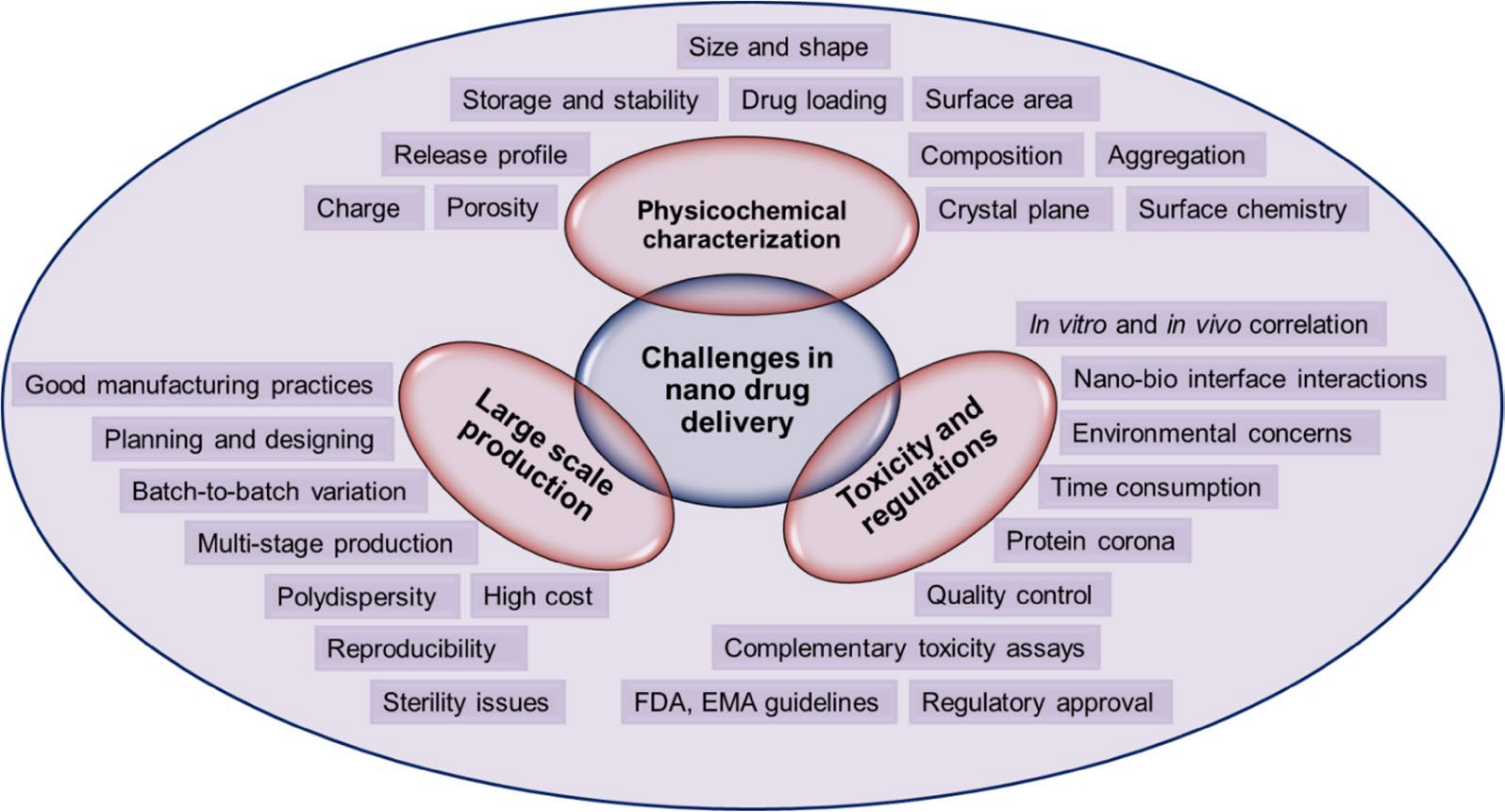
Schematic illustration representing various challenges involved in the delivery of cancer nanotherapeutics

The use of diverse nanomaterials with desired properties and recent progress in the drug delivery arena have revealed outstanding challenges in cancer therapy and management. It is anticipated that the nanomaterials will revolutionize the entire health care system based on the dramatic developments made in drug delivery sector over the past few decades. However, the design of effective cancer nanotherapeutics remains a great challenge, and only a few nanoformulations have entered clinical trials. A schematic representation of the major challenges in the delivery of cancer nanotherapeutics is depicted in Fig. 9. The physicochemical properties of nanomaterials play a significant role in the biocompatibility, and toxicity in the biological systems [284, 285]. Therefore, synthesis and characterization of the nanomaterials for drug delivery need to be carefully performed to avoid the potential unwanted toxicity of nanocarriers to healthy cells [23]. Additionally, since these nanocarriers interact with the biomolecules and may tend to aggregate forming a protein corona, disturbing the regular function of nanomedicine formulations and rendering them ineffective in controlling the cancer cell growth [286]. In conjunction to physicochemical properties, the nanomaterial storage and stability may also have an influence on their pharmacological performance [287, 288].

Another challenge in drug delivery is the safety for human health, as issues may be associated with nanomaterial, and may not have immediate impact or may not be noticeable quickly. The use of nanocarriers in the treatment of cancer may result in unwanted toxicity through unfavourable interactions with biological entities [289]. Several studies have revealed the detrimental properties of nanocarriers due to their toxicity [290, 291]. Therefore, ‘Nanotoxicology’ a branch of nanomedicine has emerged as an essential field of research, paving the way for the assessment of toxicity of nanoparticles. In addition to all the above, a significant setback in nanomedicine commercialization is the clinical translation due to the lack of in-depth understanding of nano-bio interfacial interactions. Specifically, the lack of in vitro/in vivo correlation of drug release profiles is a major lingering issue. Furthermore, the manufacturing of nanomedicine products for commercialization is a key obstacle, as large scale-production is technically challenging. Generally, only small quantities of nanomedicine are used for pre-clinical and clinical trial studies. The large-scale production of nanoformulations, however, is quite challenging as their physicochemical properties may vary from batch to batch. Moreover, the involvement of complicated multi-stage processes of production of nanotherapeutics and the high cost of raw materials renders these nanotherapeutics an expensive option. Consequently, the use of well-planned and -designed manufacturing processes are essential, and the clinical benefit must be huge which can justify the manufacturing costs.

Another key issue is the challenge of regulatory approval of nanomedicines, as there are no specific guidelines set by FDA for the products with nanomaterials. Currently used criteria have been borrowed directly from guidelines pertaining to bulk materials. The regulatory verdicts on the nanoformulated drugs are based on the individual assessment of paybacks and perils, making evaluations a time-consuming affair that causes delays in commercialization. Also, difficulties in the approval will tend to increase due to the development of multifunctional nanoplatforms. Thus, to mitigate the problems associated with nanomaterial-based therapeutic agents for cancer treatment, design and development strategies need to be employed before they are used in medicine for better treatment and human life. Understanding the complications involved in cancer cell physiology and the tumor microenvironment, along with drug and carrier pharmacokinetics is essential for the development of successful new cancer therapeutics. Alongside, case-by-case basis investigations are required to harness the tremendous potential of cancer nanotherapeutics. A comprehensive set of guidelines for regulatory approval is urgently needed to expedite the evaluation and approval of cancer nanotherapeutics.

## Conclusions

Progress in materials science and nanotechnology have brought nanomaterials-based formulations/drugs to the forefront of medical research, emerging as potential tools for cancer treatment and management. The smart design and synthesis of a library of nanomaterials, precise control over their physicochemical properties and ease of their surface functionalization to increase specificity is indeed necessary for the success of cancer nanotherapeutics. An understanding of nano-bio interfacial interactions and targeting of nanoparticles to the tumor cells is essential for cancer therapy and management. All these strategies can reduce the systemic toxicity at the tumor sites by ensuring that healthy cells are not affected. Also, several nanoplatforms have already been developed to release the cargos in response to various stimuli, offering multifunctionality and specificity. Despite the numerous advantages of the nano-based cancer therapeutics, clinical translation of these nanomedicines remains to be a challenging mission. Due to the lack of understanding of toxicity and in vivo behaviour of nanoformulations, clinical trials are experiencing major setbacks. Therefore, only a few numbers of nano-drugs available in market for the treatment of cancer. However, further advancements in nanomedicine will provide breakthroughs that represent a paradigm shift in the treatment of cancer, and can significantly contribute to an improved patient outcome. At this stage, it can be envisioned that improvement in materials is possible for next‐generation nanomedicine through smart design, and new developments can provide better cancer managment strategies.

## Data Availability

The review is based on the published data and sources of data upon which conclusions have been drawn can be found in the reference list.

## Abbreviations

EPR: enhanced permeability and retention
FDA: Food and Drug Administration
PEG: polyethylene glycol
DOX: doxorubicin
IGF1: insulin-like growth factor 1
IONPs: iron oxide nanoparticles
PDX: patient-derived xenograft
MRI: magnetic resonance imaging
AMF: alternating magnetic field
EGFR: epidermal growth factor receptor
FR: folate receptor
MSNs: mesoporous silica nanoparticles
siRNA: small interfering RNA
FITC: fluorescein isothiocyanate
RITC: rhodamine B isothiocyanate
PLGA: poly(lactic-*co*-glycolic acid)
PVA: polyvinyl alcohol
PDDAC: poly(diallyldimethylammonium chloride)
PDT: photodynamic therapy
PSMA: prostate-specific membrane antigen
CTAB: cetyltrimethylammonium bromide
CNTs: carbon nanotubes
MWCNTs: multi-walled carbon nanotubes
NIR: near-IR
BSA: bovine serum albumin
SCID: severe combined immunodeficiency
GO: graphene oxide
CD: β-cyclodextrin
DEN: dendrimer
CPT: camptothecin
PpIX: protoporphyrin IX
OSCC: oral squamous cell carcinoma
MUC1: Mucin 1
Lf: lactoferrin
PLA: polylactic acid
DTX: docetaxel
TMZ: temozolomide
BBB: blood–brain-barrier
PAMAM: polyamide amine
DHA: docosahexaenoic acid
HEP-mPEG: heparin-functionalized monomethoxy PEG
